# High-Throughput Next-Generation Sequencing of the Kidd Blood Group: Unexpected Antigen Expression Properties of Four Alleles and Detection of Novel Variants

**DOI:** 10.1159/000525326

**Published:** 2022-07-26

**Authors:** Stephanie M. Vorholt, Veronika Lenz, Burkhard Just, Jürgen Enczmann, Johannes C. Fischer, Peter A. Horn, Thomas A. Zeiler, Vera Balz

**Affiliations:** ^a^Institute for Transplantation Diagnostics and Cell Therapeutics, University Hospital Düsseldorf, Düsseldorf, Germany; ^b^Institute for Transfusion Medicine, University Hospital Essen, Essen, Germany; ^c^German Red Cross Blood Service West, Hagen/Breitscheid/Münster, Germany

**Keywords:** Next-generation sequencing genotyping, Kidd blood group, Novel variants, Blood donation, Genotype-phenotype correlation

## Abstract

**Background:**

The blood supply for patients with foreign ethnic backgrounds can be challenging, as they often have blood group and HPA patterns that differ from the variants prevalent in the German population. In addition, hemoglobinopathies requiring regular blood transfusion may be more common in such populations. High-throughput genotyping tests can facilitate the identification of the most compatible blood products, thereby reducing the risk of transfusion reactions. The present study reports the results of a molecular study for the Kidd (JK) blood group. Allele frequencies and antigen prevalence data are presented for >8,000 individuals of various origins.

**Material and Methods:**

More than 8,000 blood donors were genotyped for 22 blood group systems and 5 HPA genes using an amplicon-based next-generation sequencing (NGS) approach. As part of the test system, we focused on the JK system in more detail. Double-ARMS PCR analysis was performed for the haplotype phasing of the *JK1/JK2* and two more common synonymous polymorphisms. We performed transcript analysis to detect potential alternative splice products. For a subset of samples, a comparison between serotype and red cell genotype was conducted. Allele frequencies were determined for geographically different panels of individuals.

**Results:**

We successfully genotyped the JK blood group for 99.6% of the samples. Haplotype phasing revealed 96 different alleles. For several alleles that carry one of the synonymous SNVs c.588A>G and c.810G>A, we could not confirm the reported JK phenotypes. We found a higher frequency of JK:1 alleles for all populations except Iraqis. JK*01W.01 alleles were more common in the Asian groups and sub-Saharan Africans. A variant of the allele JK*02N.01 was present exclusively in Southeast Asians.

**Conclusion:**

Genotyping for JK antigens with a targeted NGS assay can easily be performed in routine. The interpretation that c.588A>G leads to a weak phenotype and c.810G>A to a null phenotype is questionable. IDs as well as the descriptions of alleles carrying these SNVs should be revised in the ISBT JK table.

## Introduction

The main task in transfusion medicine is to provide suitable blood products for each patient on time. This can be a challenge for people with foreign ethnic backgrounds, as they may have rare blood group phenotypes that do not match with those of people of Western European origin. The number of patients suffering from hemoglobinopathies such as sickle cell anemia or thalassemia who rely on a regular blood supply has increased significantly since 2015, when larger numbers of people from Arab countries, particularly Syria, Iran, Iraq, and the Maghreb countries, began migrating. In addition, communities from Turkey and Eastern European countries live in Germany. In order to characterize more specifically the variability of blood group genes in these populations, we designed a targeted amplicon-based next-generation sequencing (NGS) approach for genotyping 22 different blood group systems comprising 26 different genes among the 43 blood group systems currently recognized by the International Society of Blood Transfusion (ISBT) (http://www.isbtweb.org) [1]. In addition, multiple human platelet antigens are genotyped simultaneously (online suppl. Table S1; see www.karger.com/doi/10.1159/000525326 for all online suppl. material). In this report, we focus our attention on the Kidd blood group system.

The JK blood group (JK; ISBT 009) system consists of three antigens: JK1, JK2 (or Jk^a^ and Jk^b^), and JK3. The JK1 and JK2 antigens result from a single-nucleotide variant (SNV) at c.838G>A (p.Asp280Asn, rs1058396). In addition, the ISBT lists more than 50 alleles that encode altered or suppressed expression of JK antigens (http://www.isbtweb.org/fileadmin/user_upload/_ISBT_009__JK_blood_group_alleles_v6.0_01-MAR-2020.pdf). The Kidd blood group system has been known as clinically important because antibodies to JK antigens can cause immediate hemolytic transfusion reactions and hemolytic disease in neonates. They are a common cause of delayed transfusion reactions [2]. About 12% of alloimmunized patients with sickle cell disease and 4% of patients with other diseases develop antibodies to JK antigens [3, 4, 5].

This study aimed to develop a targeted NGS approach for the analysis of different blood groups and platelet antigens. Here, we provide and discuss the results of a molecular study for the JK blood group system, including SNVs, haplotypes, alleles, and frequencies, in >8,000 individuals of various origins. In addition, an investigation of genotype-phenotype correlation was performed in a subset of samples.

## Material and Methods

### Sample Cohort

The German Red Cross Blood Service recruited 7,564 individuals for blood donation who came mainly from Syria, Turkey, Iran, and Eastern European countries. The donors presented at regular blood collections at the German Red Cross Blood Service West in North-Rhine Westphalia. We further included 499 blood samples from a random cohort of blood donors from the Institute for Transplantation Diagnostics and Cell Therapeutics at the University Hospital Düsseldorf, Germany. Donors declared their country of origin in a voluntary manner (*n* = 5,829). 2,217 individuals did not provide information about their mother countries. Samples were grouped in various panels according to the geographical area of their mother countries to determine the respective allele frequencies (Table 1).

### DNA Extraction

The white blood cells remaining in the blood bag after the separation of the erythrocytes were used as starting material. The samples were diluted at 1:2 with PBS. Subsequent DNA extraction was performed using the DNAQiamp 96 DNA Blood Kit (Qiagen, Hilden, Germany) according to the manufacturer‧s instructions. Deviating from the instructions, the lysis time was extended to 30 min.

### High-Throughput Amplicon-Based NGS Assay

#### Primer Design

For high-throughput typing of blood group antigens, we chose an amplicon-based approach using Illumina technology. The NGS assay targets 22 different blood group systems and five human platelet antigen genes (online suppl. Table S1). Primer design was performed using the online tool Primer3 [6]. Primers were designed to yield amplicons ranging in size from 295 bp to 511 bp and checked for specificity and additional SNVs using SNPCheck software (https://genetools.org/SNPCheck/snpcheck.htm). Each primer pair was controlled for specific amplification using Sanger sequencing. Primers were purchased from Biolegio (Nijmegen, The Netherlands). Primers used in the NGS assay are described in online supplementary Table S2. Regarding the JK blood group system, the entire coding region (exons 3−10) including adjacent parts of the 5′-UTR, introns, and 3′-UTR of the *SLC14A1* gene was analyzed.

### NGS and Workflow

The entire set of fragments was amplified in four multiplex PCR reactions. The reaction mixture for each sample consisted of 5 μL of KAPA2G Fast Multiplex Mix (Kapa Biosystems, Cape Town, South Africa), 1 μM of each primer mix, and 20−50 ng of genomic DNA in a total volume of 10 μL. The PCR protocol was performed as follows: 95°C-3 min; 30 cycles of 95°C-15 s, 63°C-2 min, 72°C-45 s; 72°C-3 min. Further processing was done as described previously except that 190 samples were pooled [7].

For verification and proof of reproducibility, 95 samples were analyzed in three different NGS runs. Depth of coverage, quality of reads, and genotyping results were compared.

### Evaluation Criteria

Runs that were included in this study had to pass the following criteria: cluster density between 800 and 1,300 k/mm^2^, more than 70% of clusters pass the filter, and Q30 score must be higher than 70%.

### Data Analysis

Data analysis was performed using the previously described, self-developed, and accredited analysis software Blood Group_Analyzer (Institute for Transplantation Diagnostics and Cell Therapeutics, University Hospital Düsseldorf, Düsseldorf, Germany) [8]. After an error correction step, the final allele determination was performed by forming intersections of allele groups for all eight exons. Thresholds for minimum coverage were determined empirically by comparing genotyping and serotyping results for the entire set of validation samples. The minimum coverage for a fragment was set at 80 in the case of a homozygous allele group and 30 for each allele in a heterozygous allele group. Genotyping results were accepted if the appropriate depth of coverage was reached for the entire set of fragments.

### JK-Specific Analysis

#### Reference Sequences

Enumeration of exons is based on the reference sequences for allele JK*01 (Acc. No. NG_011775.4 and NM_015865.7 for genomic DNA and mRNA, respectively).

### Haplotype Phasing by Double Amplification Refractory Mutation System (Double-ARMS)

We performed a double-ARMS test to assign the two common SNVs c.588A>G and c.810G>A to JK*01 and JK*02. Similar to Lo et al. [7], we used allele-specific primer pairs to resolve the different haplotypes. The primer combinations of the haplotype-specific PCR reactions are listed in Table 2. Amplicon sizes of fragments from c.588 to c.810 and from c.810 to c.838 were 2,773 bp and 281 bp, respectively. A 506 bp fragment encompassing exon 11 of the *ABCG2* gene was co-amplified in each reaction for amplification control. The reaction mixture contained 20−50 ng of genomic DNA, 0.5 μM of each JK primer, 0.05 μM of each control primer, 0.2 mM of each dNTP, 0.25 U LongAmp Taq, and 2 μL 5× LongAmp Buffer (New England Biolabs, Frankfurt/Main, Germany) in a final volume of 10 μL. The following amplification conditions were used: 95°C-3 min; 3 cycles of 95°C-15 s, 70°C-20 s or 3.5 min; 3 cycles of 95°C-15 s, 68°C-45 s, 72°C-20 s or 3.5 min; 35 cycles of 95°C-15 s, 66°C-20 s or 3.5 min; 72°C-10 min. The elongation time of 3.5 min was used for amplification of the 2,776 bp amplicon of reaction A, and an elongation time of 20 s was used for the amplification of the 281 bp amplicon of reaction B. Phasing of other SNVs concerning *JK1/JK2* polymorphism was inferred from the presence of samples homozygous for *JK1* or *JK2*, obtained from the Erythrogene.com database [9] or analyzed in double-ARMS tests (data not shown).

### JK Transcript Analysis

RNA was isolated from reticulocytes using the Maxwell CSC RNA Blood Kit (Promega, Walldorf, Germany) or the Quick RNA Whole Blood Kit (Zymo Research Europe, Freiburg, Germany). JK-cDNA was amplified with primers targeting the exon 5−exon 6 and the exon 8−exon 9 boundaries according to the following protocol. In a reaction volume of 10 μL, 50 ng RNA was mixed with 50 pmol sense and reverse primer, each. The mixture was incubated for 5 min at 70°C and 5 min at 20°C. After the addition of 5 μL Scriptum standard reaction mix and 0.4 μL Scriptum standard enzyme (both; Bio&Sell, Feucht, Germany), cDNA fragments were amplified at 95°C-5 min; 40 cycles of 95°C-10 s, 63°C-20 s, 72°C-1 min; 72°C-2 min. Amplicons were subjected to Sanger sequencing analysis using standard protocols. Primers for cDNA amplification and Sanger sequence analysis are given in Table 2.

### Inference of JK RBC Phenotypes

For the inference of RBC phenotypes, alleles were classified into two different categories.

#### Referenced ISBT Alleles

The JK RBC phenotype of alleles was assigned according to the phenotype obtained from the ISBT website (http://www.isbtweb.org/fileadmin/user_upload/_ISBT_009__JK_blood_group_alleles_v6.0_01-MAR-2020.pdf).

#### Nonreferenced Alleles

For alleles carrying novel SNVs, we abandoned assuming a RBC phenotype because uncharacterized mutations might alter the antigenicity of the background allele.

### Serotyping of JK Red Blood Cell Antigens

The presence of JK1 and JK2 antigens was identified by RBC phenotyping for 551 randomly selected samples. In addition, the agglutination intensity was determined in 251 samples.

The first serological assay was performed on erythrocytes using an automated system (Erytra, Grifols, Leipzig, Germany) with anti-Jk^a^ and anti-Jk^b^ monoclonal antibodies (MS-15 and MS-8, respectively). For a second determination, serotyping was performed using the standard tube agglutination method with polyclonal antibodies (ND-Diagnostik, Sinsheim, Germany, and Bio-Rad, Neuberg, Germany, respectively). According to the manufacturer‧s instructions, one drop of cell suspension was mixed with one drop of reagent. The mixture was centrifuged at 800 *g* for 1 min and incubated at 37°C for 15−30 min. Agglutination was counted as Jk^a^ or Jk^b^ antigen positivity. Positive and negative control cells and Coomb‧s control cells were used for quality controls.

### Provisional Nomenclature of Novel Alleles

**Fig. 1:**
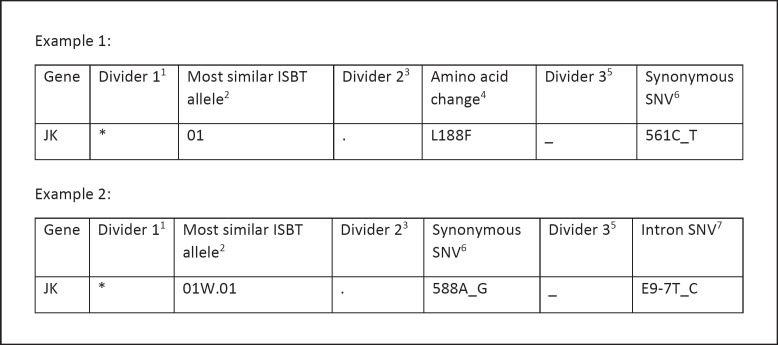
Provisional nomenclature of novel alleles. ^1^Divider 1 (*) indicates a result that was obtained using molecular typing methods. ^2^The most similar referenced ISBT allele as published by the ISBT working party [1]. ^3^Divider 2 is always a “.”. It indicates that additional amino acid changes or nucleotide changes are present. ^4^Amino acid changes are listed in consecutive order. They are termed in one-letter code with the position of amino acid in the middle. ^5^Divider 3 “_” is used to separate additional amino acid changes or nucleotide changes in the cDNA or changes which are present in the intron. ^6^Synonymous SNVs are termed as nucleotide position in cDNA immediately followed by wild-type nucleotide and changed nucleotide separated by “_”. ^7^In the case of SNVs in intron regions, the nearest exon is given followed by the distance to the exon in counts of nucleotide. +, Nucleotide change is downstream of the exon. −, Nucleotide change is upstream of the exon.

For nonreferenced alleles, a provisional nomenclature was chosen that unambiguously identifies the amino acid and nucleotide changes in the coding region as well as in intron regions (shown in Fig. 1).

### Statistics

Absolute allele frequencies were determined by direct counting of alleles according to the Hardy-Weinberg ratio. For homozygous genotyping results, alleles were counted twice. The percentage allele frequencies were calculated from the number of homo- and heterozygous samples. Typing failures (no calls) were not considered in the frequency calculation.

## Results

We conducted a targeted NGS analysis for the determination of multiple blood groups and platelet antigens in >8,000 blood donors from different origins. Because detailed results for the entire set of genes are voluminous and complex, further analysis results are described for the JK blood group system, only.

## Data Quality and Metrics

### Results for the JK Blood Group System

#### Validation Sample Set

We performed initial genotyping and serotyping for 551 randomly selected samples. These samples were used (1) to optimize the assay in terms of data quality and metrics, (2) to adapt the BloodGroup_Analyzer software for the automatic determination of genotypes, and (3) to validate the obtained genotyping results by direct comparison with erythrocyte serotypes.

#### Data Quality and Metrics

The mean depth of coverage varied between various JK amplicons (300.6−873.59 and 166.44−450.69 for homozygous and heterozygous samples, respectively) (online suppl. Table S2). In addition, depending on the quality of DNA, we observed low coverage for all amplicons for some samples. For heterozygous fragments, the proportion of both alleles was near 1:1 (online suppl. Table S2; online suppl. Fig. S1).

To prove reproducibility, a series of 95 samples were analyzed in three different NGS runs. The depths of coverage were comparable, and identical genotyping results were obtained (online suppl. Table S3; online suppl. Fig. S1). Next, we trimmed the BloodGroup_Analyzer software for automatic SNV detection and allele determination. We analyzed the depth of coverage for heterozygous and homozygous fragments for the entire set of 551 validation samples and empirically determined the minimum depth of coverage required for reliable results. The minimum coverage was set to 80 in the case of a homozygous fragment and 30 for each allele in a heterozygous allele group.

### Sequencing Data

#### Observed SNVs

The entire set of 551 samples was successfully genotyped. We detected several SNVs with the most common variants c.838G>A (p.Asp208Asn, *JK1/JK2*), c.588A>G (synonymous), c.130G>A (p.Glu44Lys), and c.810G>A (synonymous) (Table 3).

#### Haplotype Phasing of SNVs

The haplotype phase of the observed SNVs was then determined by applying a double-ARMS approach (shown in Fig. 1a). The common SNVs c.588A>G (rs2298718) and c.810G>A (rs17675299) were present in 209 and 32 *JK1/JK2* heterozygous samples, respectively. These SNVs are thought to attenuate or suppress JK expression regardless of the background allele. Eighteen samples were tested for haplophasing SNV c.588A>G to *JK1* and *JK2*, and thirteen samples were tested for all three SNVs. All *JK2* alleles showed linkage to c.588G. Among the *JK1* alleles, c.588A is more common than c.588G, except for alleles that also carry c.130G>A. The c.810G>A change was found exclusively in haplotype phasing to c.588G and c.838A.

#### Transcript Analysis

**Fig. 2:**
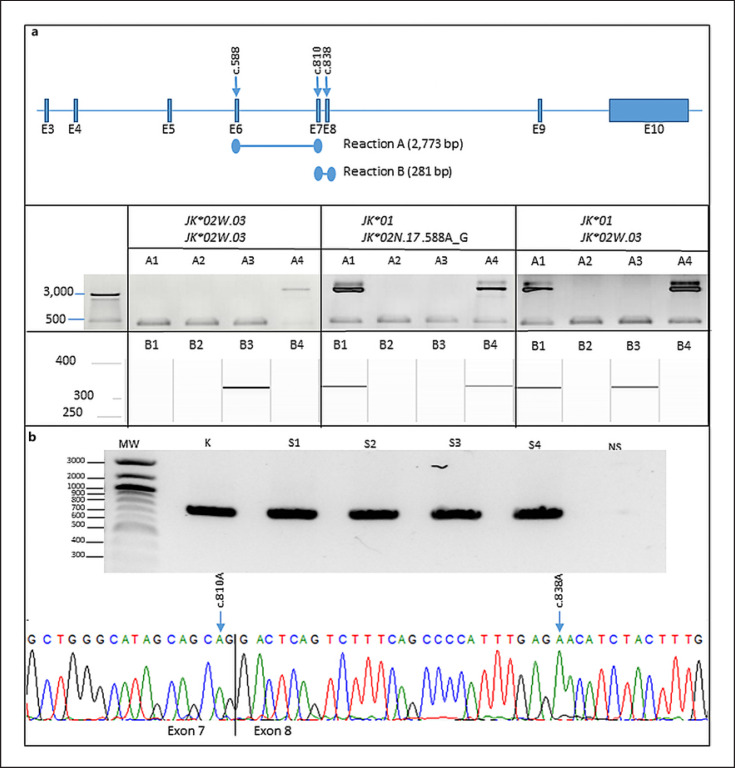
Haplotype phasing and transcript analysis. **a** Upper panel: Schematic representation of the *SLC14A1* gene. Arrows indicate SNV positions c.588, c.810, and c.838 (*JK1/JK2* SNV). Haplotype phasing of the indicated SNVs is resolved by double-ARMS reactions A and B. Lower panel: Three samples representing different genotypes containing the SNVs c.588A>G, c.810G>A, and c.838G>A were analyzed. Detection of reaction A was performed with agarose gel electrophoresis, whereas reaction B was analyzed using a QIAxcel device (Qiagen). A 506 bp fragment comprising exon 11 of the *ABCG2* gene was co-amplified in reactions A1 to A4 for amplification control. A1; c.588A/c.838G, A2; c.588G/c.838G, A3; c.588A/c.838A, A4; c.588G/c.838A, B1; c.810G/c.838G, B2; c.810A/c.838G, B3; c.810G/c.838A, B4; c.810A/c.838A. **b** Upper panel: Amplificates of Kidd cDNA. MW, molecular weight marker; NS, no sample control; K, control sample comprising a homozygous JK*01 genotype; S1 to S4, homozygous samples for c.810A. Lower panel: Sanger sequence analysis of cDNA of a c.810A homozygous sample. Arrows indicate nucleotides c.810A and c.838A. The exon border of exon 7 to exon 8 is shown. No aberrant splicing products are observed.

Because the SNV c.810G>A affects the penultimate position of exon 7, it might disturb proper splicing. Therefore, we performed a transcript analysis of four homozygous c.810A samples. Exons 6−8 of the *JK* transcript were amplified and subjected to Sanger sequencing analysis. We did not observe alternative splice products suggesting that a full-length JK transcript is present (shown in Fig. 2b).

#### Allele Determination

Haplotype phasing resulted in a total number of sixteen different alleles present in the validation sample set. Of these, six alleles were already referenced by ISBT. The remaining alleles are variants of alleles JK*01, *01W.01*, *02W.03*, *02N.01*, and *02N.17* and include an additional missense or synonymous variant (Table 3). For nonreferenced alleles, a provisional nomenclature was chosen that uniquely identifies the amino acid and nucleotide changes (shown in Fig. 1).

#### Inference of RBC Phenotypes

Next, we inferred the RBC phenotypes from genotyping results. For ISBT-referenced alleles, the phenotype was taken from the JK ISBT allele table (http://www.isbtweb.org/fileadmin/user_upload/_ISBT_009__JK_blood_group_alleles_v6.0_01-MAR-2020.pdf). For nonreferenced alleles with synonymous SNVs other than c.588A>G and c.810G>A, and novel missense variants, we refrained from assuming an RBC phenotype (Table 3).

#### Comparison of Inferred RBC Phenotypes and Serotypes

We then compared the inferred RBC phenotypes with experimentally determined serotypes (Table 3). Only samples that were either heterozygous for *JK1/JK2* or homozygous for a given allele were considered. In this way, phenotypes of fourteen different alleles could be analyzed. Agglutination intensity was used as a measure of the degree of antigen expression. Inferred phenotypes of JK:1, JK:1^weak^, and JK:−2 were identical to serotypes for alleles JK*01, *01W.01* (±c.588G), and *02N.01.588A_G*, respectively. The two synonymous SNVs c.588A>G (rs2298718) and c.810G>A (rs17675299) occur at high frequencies of 0.6016 and 0.0517, respectively. These variants were recorded to be associated with attenuated (c.588G) and absent (c.810A) JK antigen expression regardless of whether they are present on a *JK1* or *JK2* allele. For JK*01W.03 and *02W.06* alleles, which both carry the c.588G variant, diminished antigen expression would have been expected, while agglutination intensities of 3−4 were observed (5 and 305 samples, respectively). Alleles JK*02N.17 with c.810A and JK*01N.20 were both expected to be JK-seronegative but were typed as JK:2 and JK:1, respectively, with high antigen expression regardless of the homozygous or heterozygous presence of *JK1/JK2*. Altogether, neither both synonymous SNVs nor the amino acid and nucleotide changes present in allele JK*01N.20 appear to alter the antigenicity of the JK protein.

#### Serotyping of Novel Variants

We detected two JK*02W.03 alleles with additional synonymous SNVs, c.582C>T and c.1095T>C (1 and 13 samples, respectively). Serotyping revealed JK:2 expression for all samples. In the case of c.1095T>C, an agglutination intensity of 4 indicated a high expression level. In addition, four alleles carried nonreferenced missense variants. JK*01.Q21R was found in a heterozygous sample expressing JK:1, while JK*02W.03.P90S, *02W.03.M167*V, and *02W.03.G325S* were found similarly in JK:2 positive samples (Table 3). Overall, none of these nonreferenced SNVs appears to change the JK1/JK2 antigenicity. Since allele JK*01.L148F was found in combination with JK*01, we were not able to determine the RBC serotype.

#### Discrepant Results

Five false serological RBC phenotype determinations were observed (0.91%, online suppl. Table S4). Double-ARMS tests of newly isolated DNA confirmed NGS genotyping results for all samples. Thus, for these samples, we assume sample mix-ups.

### Prospective Data Set

#### Data Quality

A cohort of 7,512 additional samples derived from blood donors of a different ancestry was analyzed using the NGS assay. Of these, 7,482 (99.6%) were successfully genotyped for the JK blood group system. The remaining 30 samples did not provide a clear genotyping result. Due to poor sample quality, one or more exons did not reach the required depth of coverage (Table 1).

### General Sequencing Data

#### Observed SNVs

Besides the SNVs already described for the validation sample set, we detected a large number of SNVs scattered throughout the coding region and adjacent parts of the 5′-UTR, introns, and 3′-UTR for both *JK:1* and *JK:2* alleles (Table 4).

### Haplotype Phasing and Allele Determination

Haplotype phasing revealed 95 different alleles, including all alleles already discovered for the validation sample set, except JK*02N.17 (Table 4). Two missense SNVs were discovered in multiple alleles, p.Met167Val and p.Val175Ile (3 and 2 alleles, respectively). Although most variants have been reported previously in the gnomAD database [10], 26 variants are described for the first time. These variant alleles were submitted to the GenBank database (https://www.ncbi.nlm.nih.gov/gene). Accession numbers are given in Table 4.

### Allele Frequencies in Various Blood Donor Panels

Genotyping data from the validation and prospective data sets were combined to determine allele frequencies. A summary of genotyping results is reported in online supplementary Table S5.

The majority of individuals carry at least one of the alleles JK*01, 01W.01 (±c.588G), *02W.03*, and *02N.17.588A_G*, regardless of country of origin (8,006 samples, 99.7%) (Table 5). The frequencies of *JK1* and *JK2* alleles are nearly equal for most of the blood donor panels (Table 5 and online suppl. Table S6). Deviations from this distribution were observed in sub-Saharan Africans with a higher frequency of *JK1* alleles (71.43%), which is consistent with previous data (73.45% and 72.88% for gnomAD and ALFA database, respectively) [10, 11]. In contrast, we found a slightly higher frequency for *JK2* alleles in Iraqis (52.09%), but this deviation was not significant (*p* > 0.05). The most common weak allele was JK*01W.01 and variations thereof. It was more frequent in Asians (25.21−33.12%) and people from sub-Saharan Africa than for other populations (20.71 vs. 9.44−12.82%) confirming the frequency data given in the databases gnomAD (19.75% for Africans/African Americans and 26.78−40.58% for Asians) and ALFA (19.53% for Africans/African Americans and 25.16−39.16% for Asians). Other weak alleles, JK*01W.02, 01W.04, and *02W.04* and variations, were rare (in total 0.13%). The novel allele JK*02W.03.M167V was found mainly in individuals from Southeast and East Asia (16.67% vs. 0.06−0.81% in other populations). This is consistent with the gnomAD database, where the underlying SNV c.499A>G was found in 18.95% of East Asian samples but rarely in other populations (max. 0.09%). ALFA database lists a frequency of 19.1% for East Asians and less than 0.3% for non-Asians. Three SNVs lead to translation arrest upstream of the *JK1/JK2* determining amino acid. These variants were considered as JK:−1,−2. In addition, the variant p.Arg64Gln was previously referenced to abolish JK:2 expression in allele JK*02N.09. Here, we found the same variant coupled to JK:1, suggesting the allele JK*01.R64Q to be silenced as well. Taking this into account, null alleles were present 25 times, including JK*01N.09, 01N.18, 02N.01, 02N.05, 02N.06, 02N.08, and *02N.09*, as well as the new alleles JK*01.163del, 01.R64Q, 02W.03.Y37X, and *02W.03.56insA_549G_A* (0.15% in total).

The null allele JK*02N.01.588A_G was present only in Southeast Asians (Philippines, Thailand, and Vietnam). In addition, one Filipino was homozygous for this allele. In gnomAD and ALFA databases, this allele was found in 1−2% of Asians.

## Discussion

Transfusion therapy remains the mainstay of treatment for patients with hemoglobinopathy. The development of red cell alloantibodies is a serious and common complication, as it increases the risk for hemolytic transfusion reactions and occasionally life-threatening events [12]. In addition, delays in the identification of compatible red cell units may occur. While only 2−5% of the general population develop allo-RBC-antibodies after transfusion therapy, the prevalence of alloimmunization for patients with sickle cell disease ranges from 5 to 75% [13, 14, 15].

To ensure transfusion safety, blood donor samples are routinely tested for the major antigens of the Rh, ABO, and Kell blood group systems. However, several clinically important alloantibodies to other high-frequency antigens have also been identified. Therefore, extended RBC antigen matching was recommended for patients at risk for alloimmunization [16]. Because extended blood group typing by serology is not applicable for high sample numbers, especially when rare typing sera are not available, prospective high-throughput blood donor screening for expanded blood group panels might improve the provision of blood products for patients with rare phenotypes or patients immunized against common antigens. In the present report, we used an amplicon-based NGS approach for genotyping several blood groups and platelet antigen genes including the Kidd blood group system. Allo-JK antibodies can cause immediate hemolytic transfusion reactions, neonatal hemolytic disease, and delayed transfusion reactions [2]. It was recommended to administer JK-matched red cell units to immunized patients [16]. Here, we turn the attention to JK blood group genotyping. We provide molecular data of >8,000 blood donors of various origins. Moreover, for a subset of samples, we performed genotype-phenotype correlation.

### Discordance in the Description of Alleles − Consequences for Nomenclature

For a subset of 551 samples, we inferred the RBC phenotype based on the specifications given by ISBT in the current table for the JK blood group system (http://www.isbtweb.org/fileadmin/user_upload/_ISBT_009__JK_blood_group_alleles_v6.0_01-MAR-2020.pdf) and compared the results to experimentally obtain serotypes. We demonstrated that neither of the frequent synonymous SNVs c.588A>G and c.810G>A lead to altered JK antigen expression. Moreover, we were able to exclude disruption of proper transcript splicing caused by c.810A as previously suspected [17, 18]. Similarly, all samples carrying allele JK*01N.20 showed normal JK:1 expression. In addition, c.588G was present in all but one JK:2 expressing alleles. This nucleotide change was initially described to be present in the regular JK*02 allele [19]. Because the ISBT working party focuses on variants that result in a change of antigenicity, the c.588A>G change may intentionally be omitted from some allele descriptions. However, our results strongly suggest that the IDs and phenotype descriptions of alleles JK*01W.06, 01N.20, and *02N.17*, as well as JK*01N.19, which carry the same SNV as JK*02N.17, should be revised. In addition, since the c.588G variant is part of the regular JK*02 allele, JK*02W.03 should be removed and the nucleotide description of JK*02 should be revised.

### Characterization of Novel *JK* Alleles

In the present study, we discovered a large number of rare SNVs. In 2016, the Erythrogene.com database was established, describing the full coding regions of blood group alleles discovered during the 1000 Genomes project [9]. This simplified haplotyping of some nonreferenced alleles but no unique allele names was given. HLA typing is routinely performed by NGS, and the sequencing results obtained are compared with a reference database in which alleles are recorded with unique IDs, regardless of whether protein characteristics are affected (IPD-IMGT/HLA database, available at https://www.ebi.ac.uk/ipd/imgt/hla/). Multiple nonreferenced alleles are often found during genotyping by sequence analysis assays. Here, a streamlined protocol for naming novel alleles and a database analogous to the IPD-IMGT/HLA database in which the nucleotide sequences of these alleles are recorded would be helpful. However, the clinical significance of those novel alleles is difficult to predict without clinical correlation. Further functional studies are needed to definitively determine whether a variant results in altered expression or encodes a previously undescribed blood group antigen.

### Classification of Weak and Null Alleles by Targeted *JK* Genotyping

In our sample cohort, a large number of donors carried at least one weakly expressed or null allele (12.5% and 0.3%, respectively). In blood group genetics, the presence of alleles with attenuated or silenced antigen expression is a well-known problem. JK typing can be performed using serological RBC phenotyping or DNA-based molecular typing methods. When using antibody-based typing methods, weak antigen expression may be missed depending on the method protocol, reagents, or antibodies. In addition, serological typing of pre-transfused patients is frequently impossible [20]. A falsely negative typed blood unit may boost antibody levels in previously sensitized patients. On the other hand, molecular typing methods are often limited to the identification of the *JK1/JK2* polymorphism [21, 22, 23, 24, 25, 26, 27]. In donor typing, the risk of false-positive results is negligible as they are phenotypically homozygous. Therefore, they are unlikely to harm the recipient if transfused to a heterozygous patient.

### Allele Frequencies in Different Populations

Consistent with several other reports and databases [10, 11, 28, 29, 30, 31, 32], we found a nearly equivalent frequency of *JK1* and *JK2* alleles for most of the blood donor panels and an elevated frequency of *JK1* alleles in sub-Saharan Africans confirming other studies [10, 11, 32]. Similarly, the higher frequencies of JK*01W.01 and its variants for Southeast and Eastern Asian populations as well as sub-Saharan Africans have been described before [10, 11].

The frequency of the novel allele JK*02W.03.M167V was increased in the Eastern Asian group. However, we serotyped one JK*02W.03.M167V homozygous sample and four samples carrying this allele combined with a *JK1* allele for JK:2 expression. Our results suggest that this missense variant does not have an impact on JK:2 antigen expression.

### Benefits for Blood Banking Services

The described assay has the potential to allow for transfusion of matched blood products to the patient‧s phenotype. Thus, it could prevent adverse transfusion reactions leading to increased transfusion-related mortality. In addition, it will improve transfusion safety by identifying the most compatible blood products, thereby reducing the risk of alloimmunization. The method makes it possible to test a large number of donors without concerns of expense or lack of reagents, thus ensuring a sufficient supply of typed blood donors. Currently used DNA-based methods for the *JK1/JK2* polymorphism will not identify the rare JK:−1,−2 blood donors because the mutations that cause loss of antigen expression are located in other parts of the *SLC14A1* gene. In contrast, our assay is based on the analysis of the entire coding sequence including parts of the adjacent introns, thereby allowing for the identification of those rare JK-negative blood donors.

In addition, besides the high accuracy and high throughput, it has the advantage of large flexibility by combining the analysis of multiple clinical important red cell antigens. The failure rate of 0.37% across the entire sample set was very low, strongly suggesting the feasibility of this novel assay for routine diagnostics. As we did not find a major variance in *JK1/JK2* allele frequencies between different panels of blood donors, except for sub-Saharan Africans, the supply of JK-matched blood products can be sufficiently performed by the blood banking services in European countries.

### Limitations

This study has some limitations. Serotyping data are lacking for the majority of samples. Here, haplotype phasing was performed using SNV frequencies from databases. Although this strategy is helpful and accurate in most cases, it is risky in a clinical context when phenotyping data are not available. The clinical significance of new alleles is difficult to predict without clinical correlation. Further functional studies and appropriate RBC serotype identification are of particular importance to determine the impact of novel missense variants. In addition, because of the amplicon-based approach of the NGS assay, mutations present in parts of the *SLC14A1* other than the coding region and adjacent intron sequences may be missed and may lead to attenuation or suppression of JK expression.

## Conclusions

Overall, we present a targeted amplicon-based NGS workflow that is suitable for high-throughput genotyping of the Kidd blood group. Rare weak and null alleles that may be missed by currently used serotyping and molecular genotyping assays were reliably detected. The combined strategy of NGS-based genotyping and serological RBC phenotyping revealed conflicting expression characteristics for several ISBT-assigned alleles. Particularly, we show that the interpretation that c.588A>G leads to a weak phenotype and c.810G>A to a null phenotype is questionable. We thus recommend that the IDs, as well as nucleotide and phenotype descriptions of those alleles carrying one of these synonymous SNVs, be revised. A streamlined protocol for naming unreferenced alleles in conjunction with a detailed database of allele sequences and, if available, phenotype descriptions would be helpful for further studies.

## Statement of Ethics

In the opinion of the Ethics Committee of the University Hospital Düsseldorf, approval of the study was not required. The donor consents in writing to the donation process and all necessary tests before the start of the donation, including genetic testing. Serological determination and, in some cases, molecular genotyping of blood group antigens are among the required tests before transfusion of blood products.

## Conflict of Interest Statement

The authors declare that they have no conflict of interest.

## Funding Sources

This study was funded by the European Regional Development Fund 2014−2020 (EFRE), Grant/Award No. EFRE-0800961.

## Author Contributions

S.M.V., J.E., and V.B. collected, assembled, analyzed, and interpreted the data, wrote the manuscript, and approved the final version. J.E., J.C.F., T.A.Z., P.A.H., and V.B. conceived and designed the study. B.J. and V.L. provided study material and performed laboratory testing.

## Data Availability Statement

The data supporting this study‧s findings are available from the corresponding author, V.B., upon reasonable request.

## Supplementary Material

Supplementary data

Supplementary data

Supplementary data

Supplementary data

Supplementary data

Supplementary data

Supplementary data

## Figures and Tables

**Table 1:** Composite of sample cohort

Panel	Validation samples (*n* = 551)	Prospective samples (*n* = 7,512)	No calls (*n* = 30)	Total genotyped samples (*n* = 8,033)
OTH	182	2,036	10	2,207
TUR	140	2,148	8	2,280
EEKCA	63	788	2	849
SYR	48	661	3	706
IRAN	24	305	1	328
SAS	12	226	0	238
NAF	14	215	2	227
ARPE	16	180	1	195
IRAQ	14	153	0	167
SEEA	13	144	0	157
CSAM	4	74	0	78
SSAF	14	56	0	70
WEUR	7	528	3	531

In terms of simplified description, blood donors were grouped according to the geographical area of their mother countries. OTH, other: country of origin not specified, Cyprus; TUR, Turkey; EEKCA, Eastern Europe, Caucasus, Central Asia: Albania, Armenia, Azerbaijan, Belarus, Bosnia - Herzegovina, Bulgaria, Croatia, Georgia, Greece, Yugoslavia, Kazakhstan, Kyrgyzstan, Kosovo, Lithuania, Macedonia, Moldova, Montenegro, Poland, Romania, Russia, Serbia, Slovakia, Slovenia, Tajikistan, Ukraine, Hungary, Uzbekistan; SYR, Syria; IRAN, Iran; SAS, South Asia: Afghanistan, Bangladesh, India, Nepal, Pakistan, Sri Lanka; NAF, Northern Africa: Morocco, Egypt, Algeria, Libya, Tunisia; ARPE, Arabian Peninsula: Bahrain, United Arab Emirates, Kuwait, Lebanon, Qatar, Saudi Arabia, Israel, Jordan, Palestine; IRAQ, Iraq; SEEA, Southeast and East Asia: China, Indonesia, Japan, Korea, Malaysia, Mongolia, Philippines, Singapore, South Korea, Taiwan, Thailand, Tibet, Vietnam; CSAM, Central and South America: Argentina, Bolivia, Brazil, Chile, Costa Rica, Ecuador, El Salvador, Guatemala, Columbia, Mexico, Paraguay, Peru, Venezuela, Jamaica, Caribbean, Cuba, Dominican Republic; SSAF, Sub-Saharan Africa: Angola, Burkina-Faso, Congo, Ivory Coast, Eritrea, Gambia, Ghana, Guinea, Cameroon, Kenia, Mali, Mozambique, Nigeria, Senegal, South Africa, Togo, Uganda, Zambia, Zimbabwe; WEUR, Western Europe: Austria, England, Ireland, Italy, Netherlands, Portugal, Spain, Germany.

**Table 2:** Primers used in double-ARMS and cDNA analysis

Primer No.	Test	Name	Sequence	Orientation*	Target	Reaction
1	Double-ARMS	JK588A_S	GCCACAGGACATTACAATCCA	F	c.588A	A
2	Double-ARMS	JK588G_S	GCCACAGGACATTACAATCCG	F	c.588G	A
3	Double-ARMS	JK810A_R	AGGGCTCTTGTGCTCACCT	R	c.810A	A
4	Double-ARMS	JK810G_R	GGGCTCTTGTGCTCACCC	R	c.810G	A
5	Double-ARMS	JK810A_S	CATTGCTGGGCATAGCAGCA	F	c.810A	B
6	Double-ARMS	JK810G_S	ATTGCTGGGCATAGCAGCG	F	c.810G	B
7	Double-ARMS	JK01_R_SSP2	AGAGTCCAAAGTAGATGTC	R	c.838G	B
8	Double-ARMS	Jk02_R_SSP2	AGAGTCCAAAGTAGATGTTC	R	c.838A	B
9	Double-ARMS	JRE11S	CCAGGGACGTTGGCCAAGC	F	Amplification control	A, B
10	Double-ARMS	JRE11R	GGTGGATGTCTGTAGTCGAGGC	R	Amplification control	A, B
11	cDNA	JKRNA_F3	GCTTAAAGACAAACCCGTGGTGC	F	Exon 5/exon 6 border	
12	cDNA	JKRNA_R3	GGTGGGTTTGCCAGGTGAGC	R	Exon 8/exon 9 border	

Allele-specific nucleotides are underlined.

* F, forward; R, reverse.

**Table 3:** Genotyping and phenotyping data of validation sample set

Allele name	Background allele	Additional nucleotide change*	Additional amino acid exchange*	Observations, *n*	Heterozygous JK:1,2 samples, *n*	Allele-specific homozygous samples, *n*	Presumed RBC phenotype^||^	RBC serotype	Intensity of agglutination^§^	Minor allele frequency†	Exon/intron number	dbSNP number^‡^
ISBT-referenced alleles												
JK*01	−	−	−	433	208	80	JK:1	JK:1	3-4			
JK*01N.20$	JK*01	c.28G>A	p.Val10Met	9	1	1	JK:Null	JK:1	n.d.	0.0179	Exon 3	rs113578396
		c.226G>A	p.Val76Ile								Exon 4	rs113029149
		c.303G>A	Synonymous								Exon 4	rs28994287
		c.588A>G	Synonymous								Exon 6	rs2298718
JK*01W.01	JK*01	c.130G>A	p.Glu44Lys	2	−	−	JK:1 ^weak^	−	Not possible	0.1376	Exon 3	rs2298720
JK*01W.06$	JK*01	c.588A>G	Synonymous	11	4	1	JK:1 ^weak^	JK:1	4	0.6124	Exon 6	rs2298718
JK*02N. 17$	JK*02	c.810G>A	Synonymous	1	1	−	JK:Null	JK:2	n.d.	0.0456	Exon 7	rs17675299
JK*02W.03$	JK*02	c.588A>G	Synonymous	413	229	77	JK:2 ^weak^	JK:2	3-4	0.4208	Exon 6	rs2298718
Nonreferenced alleles												
JK*01.Q21R	JK*01	c.62A>G	p.Gln21 Arg	1	1	−	Unknown	JK:1	4	0.0003	Exon 3	rs146079238
JK*01.L148F	JK*01	c.442C>T	p.Leu148Phe	2	−	−	Unknown	−	Not possible	0.0002	Exon 4	rs563016158
JK*01 W.01.588A_G	JK*01W.01	c.588A>G	Synonymous	147	63	16	JK:1 ^weak^	JK:1^weak^ to	2-4	0.6124	Exon 6	rs2298718
JK*02N.01.588A_G	JK*02	c.342-1G>A	p.exon 5	1	1	−	JK:Null	JK:2−	0	0.0005	Intron 4	rs78937798
		c.588A>G	skipped								Exon 6	rs2298718
			Synonymous									
JK*02N. 17.588A_G$	JK*02N.17	c.588A>G	Synonymous	56	31	2	JK:Null	JK:2	4	0.6124	Exon 6	rs2298718
JK*02W.03.P90S$	JK*02W.03	c.268C>T	p.Pro90Ser	2	1	−	Unknown	JK:2	4	<0.0001	Exon 4	rs147790844
JK*02 W.03.M167V$	JK*02W.03	c.499A>G	p.Met167Val	9	4	1	Unknown	JK:2	3-4	0.0082	Exon 6	rs2298719
JK*02W.03.582C_T$	JK*02W.03	c.582C>T	Synonymous	1	1	−	Unknown	JK:2	n.d.	0.0035	Exon 6	rs34756616
JK*02W.03.1095T_C$	JK*02W.03	c.1095T>C	Synonymous	13	8	−	Unknown	JK:2	4	0.0039	Exon 10	rs28898897
JK*02W.03.G325S	JK*02W.03	c.973G>A	p.Gly325Ser	1	1	−	Unknown	JK:2	n.d.	0.0001	Exon 9	rs140320419

* Enumeration of nucleotide and amino acid changes according to reference allele JK*01.

|| RBC phenotype refers to the JKISBT allele table (http://www.isbtweb.org/fileadmin/user_upload/_ISBT_009_JK_blood_group_alleles_v6.0_01-MAR-2020.pdf).

§ Intensity of agglutination as determined in allele-specific homozygous or heterozygous *JK1/JK2* samples. Scale *0-4,* with 4 being the highest agglutination intensity. n.d., not determined; Not possible, the allele is in combination with a second allele of the same background allele.

$ Discrepancy between predicted phenotype and serotype.

† Minor SNV frequency as published by gnomAD database v3.1.1 [5].

‡ dbSNP number obtained from dbSNP database (https://www.ncbi.nlm.nih.gov/projects/SNP/).

**Table 4:** Prospective genotyping data

Allele	Background allele	Additional nucleotide change(s)*	Predictive additional amino acid change(s)*	Observations, *n*	Observed allele frequency	Minor allele frequency^§^	Exon/intron number	dbSNP number^†^	GenBank accession number
ISBT referenced alleles									
JK*01	−	−	−	5,607	0.3748	0.5792	−	−	
JK*01 N.09	JK*01	c.27_50del	p.Val10_Arg17del	3	<0.0001	0.0014	Exon 3	rs547922260	
JK*01N.18	JK*01	c.190C>T	p.Arg64Trp	2	<0.0001	<0.0001	Exon 4	rs552191 196	
JK*01N.20	JK*01	c.28G>A	p.Val10Met	125	0.0084	0.0179	Exon 3	rs113578396	
		c.226G>A	p.Val76Ile				Exon 4	rs113029149	
		c.303G>A	Synonymous				Exon 4	rs28994287	
		c.588A>G	Synonymous				Exon 6	rs2298718	
JK*01W.01	JK*01	c.130G>A	p.Glu44Lys	30	0.0020	0.1376	Exon 3	rs2298720	
JK*01W.03	JK*01	c.28G>A	p.Val10Met	1	<0.0001	0.0179	Exon 3	rs113578396	
JK*01W.04	JK*01	c.226G>A	p.Val76Ile	1	<0.0001	0.022	Exon 4	rs113029149	
JK*01W.06	JK*01	c.588A>G	Synonymous	87	0.0058	0.6124	Exon 6	rs2298718	
JK*02	−	−	−	1	<0.0001	0.4208	−	−	
JK*02W.03	JK*02	c.588A>G	Synonymous	5,941	0.3971	0.4208	Exon 6	rs2298718	
		c.838G>A	p.Asp280Asn				Exon 8	rs1058396	
Nonreferenced alleles									
JK*01.V8F	JK*01	c.22G>T	p.Val8Phe	1	<0.0001	−	Exon 3	Novel	MW604784
JK*01.Q21R	JK*01	c.62A>G	p.Gln21 Arg	7	<0.0001	0.0003	Exon 3	rs146079238	
JK*01.G27R	JK*01	c.79G>A	p.Gly27Arg	1	<0.0001	−	Exon 3	Novel	MW604779
JK*01.105T_C	JK*01	c.105T>C	Synonymous	2	<0.0001	<0.0001	Exon 3	rs1417111456	
JK*01.159C_T	JK*01	c.159C>T	Synonymous	2	<0.0001	0.0001	Exon 4	rs202168702	
JK*01.V54M	JK*01	c.160G>A	p.Val54Met	1	<0.0001	<0.0001	Exon 4	rs377124382	
JK*01.163del	JK*01	c.163del	p.Val55Cysfs*25	1	<0.0001	−	Exon 4	Novel	MW604785
JK*01.R64Q^||^	JK*01	c.191G>A	p.Arg64Gln	1	<0.0001	0.0002	Exon 4	rs114362217	
JK*01.V76A	JK*01	c.227T>C	p.Val76Ala	1	<0.0001	−	Exon 4	Novel	MW712669
JK*01.V87I	JK*01	c.259G>A	p.Val87Ile	1	<0.0001	−	Exon 4	Novel	MW604783
JK*01.G96V	JK*01	c.291G>T	p.Gly96Val	1	<0.0001	<0.0001	Exon 4	rs2047018661	
JK*01.L148F	JK*01	c.442C>T	p.Leu148Phe	6	<0.0001	0.0002	Exon 4	rs563016158	
JK*01.L188F_561C_T	JK*01	c.561C>T	Synonymous	1	<0.0001	<0.0001 <0.0001	Exon 6	rs778172038	
		c.562C>T	p.Leu188Phe				Exon 6	rs200153291	
JK*01.T308I	JK*01	c.923C>A	p.Thr308Ile	1	<0.0001	<0.0001	Exon 8	rs753184657	
JK*01.T346M	JK*01	c.1037C>T	p.Thr346Met	1	<0.0001	<0.0001	Exon 10	rs1377322159	
JK*01.M352I	JK*01	c.1056G>A	p.Met352Ile	4	<0.0001	<0.0001	Exon 10	rs1037329168	
JK*01.L364F	JK*01	c.1090C>T	p.Leu364Phe	3	<0.0001	−	Exon 10	Novel	MW604780
JK*01.1095T_C	JK*01	c.1095T>C	Synonymous	6	<0.0001	0.0039	Exon 10	rs28898897	
JK*01.V367A	JK*01	c.1100T>C	p.Val367Ala	1	<0.0001	−	Exon 10	Novel	MW604782
JK*01.N373I	JK*01	c.1118A>T	p.Asn373Ile	1	<0.0001	<0.0001	Exon 10	rs35942326	
JK*01.E4+10C_T	JK*01	c.341 + 10C>T	−	2	<0.0001	−	Intron 4	Novel	MW604801
JK*01.E5-19T_A	JK*01	c.342-19T>A	−	1	<0.0001	0.0001	Intron 4	rs199975559	
JK*01.E5-23G_A	JK*01	c.342-23G>A	−	7	<0.0001	0.0001	Intron 4	rs377318707	
JK*01.E5+ 12T_C	JK*01	c.470+12T>C	−	2	<0.0001	0.0029	Intron 5	rs114641857	
JK*01.E8+31G_A	JK*01	c.946+31G>A	−	1	<0.0001	<0.0001	Intron 8	rs1473703649	
JK*01.E9-13T_C	JK*01	c.947-13T>C	−	1	<0.0001	−	Intron 8	Novel	MW604802
JK*01.E9+18T_C	JK*01	c.996+18T>C	−	1	<0.0001	0.0001	Intron 9	rs374156279	
JK*01N.09.V175I	JK*01 N.09	c.499G>A	p.Val 175Ile	1	<0.0001	0.001	Exon 6	rs138222201	
JK*01 W.01.D11N_588A_G	JK*01W.01	c.31G>A	p.Asp11 Asn	3	<0.0001	<0.0001	Exon 3	rs1307492731	
		c.588A>G	Synonymous				Exon 6	rs2298718	
JK*01W.01.219C_T_588A_G	JK*01W.01	c.219C>T	Synonymous	1	<0.0001	<0.0001	Exon 4	rs779835840	
		c.588A>G	Synonymous				Exon 6	rs2298718	
JK*01W.01.279C T 588A_G	JK*01W.01	c.279T>C	Synonymous	1	<0.0001	<0.0001	Exon 4	rs1391927371	
		c.588A>G	Synonymous				Exon 6	rs2298718	
JK*01W.01.T127I_588A_G	JK*01W.01	c.380C>T	p.Thr127Ile	1	<0.0001	−	Exon 5	novel	MW604786
		c.588A>G	Synonymous				Exon 6	rs2298718	
JK*01W.01.516C_T_588A_G	JK*01W.01	c.516C>T	Synonymous	2	<0.0001	<0.0001	Exon 6	rs747405896	
		c.588A>G	Synonymous				Exon 6	rs2298718	
JK*01 W.01.588A_G	JK*01W.01	c.588A>G	Synonymous	1,820	0.1216	0.1376	Exon 6	rs2298718	
JK*01 W.01.588A_G_E9-7T_G	JK*01W.01	c.588A>G	Synonymous	1	<0.0001	<0.0001	Exon 6	rs2298718	
		c.812-7T>C	Synonymous				Intron 8	rs567213799	
JK*01 W.01.F329V_588A_G	JK*01W.01	c.588A>G	Synonymous	1	<0.0001	<0.0001	Exon 6	rs2298718	
		c.985T>G	p.Phe329Val				Exon 9	rs767190566	
JK*01 W.01.P205S 588A_G	JK*01W.01	c.613C>T	p.Pro205Ser	1	<0.0001	<0.0001	Exon 6	rs760632131	
		c.588A>G	Synonymous				Exon 6	rs2298718	
JK*01 W.02.402T_C_588A_G	JK*01	c.402T>C	Synonymous	6	<0.0001	0.0041	Exon 5	rs16978473	
		c.511T>C	p.Trp171 Arg				Exon 6	rs9948825	
		c.588A>G	Synonymous				Exon 6	rs2298718	
JK*01W.02.402T_C_588A_G_E5-24C_T	JK*01	c.402T>C	Synonymous	1	<0.0001	0.0041	Exon 5	rs16978473	
		c.342-24C>T	intron				Exon 5	rs2021361 16	
		c.511T>C	p.Trp171 Arg				Exon 6	rs9948825	
		c.588A>G	Synonymous				Exon 6	rs2298718	
JK*01 W.04.303G_A	JK*01 W.04	c.303G>A	Synonymous	4	<0.0001	0.0218	Exon 4	rs28994287	
JK*01W.06. V54M	JK*01W.06	c.160G>A	p.Val54Met	2	<0.0001	<0.0001	Exon 4	rs377124382	
JK*01 W.06.M 167V	JK*01W.06	c.499A>G	p.Met167Val	3	<0.0001	0.0082	Exon 6	rs2298719	
JK*02N.01.588A_G	JK*02	c.342-1G>A	p.exon 5 skipped	5	<0.0001	0.0005	Intron 4	rs78937798	
		c.588A>G					Exon 6	rs2298718	
JK*02N.05.588A_G	JK*02	c.588A>G	Synonymous	1	<0.0001	<0.0001	Exon 6	rs2298718	
		c.723del	p.Gly243Alafs*20				Exon 7	rs759505281	
		c.838G>A					Exon 8	rs78242949	
JK*02N.06.588A_G	JK*02	c.588A>G	Synonymous	1	<0.0001	0.0018	Exon 6	rs2298718	
		c.871T>C	p.Ser291Pro				Exon 8	rs78242949	
		c.838G>A	p.Asp280Asn				Exon 8	rs78242949	
JK*02N.08.588A_G	JK*02	c.588A>G	Synonymous	2	<0.0001	0.0002	Exon 6	rs2298718	
		c.838G>A	p.Asp280Asn				Exon 8	rs1058396	
		c.956C>T	p.Thr319Met				Exon 9	rs565898944	
JK*02N.09.588A_G	JK*02	c.191G>A	p.Arg64Gln	5	<0.0001	0.0002	Exon 4	rs114362217	
		c.588A>G	Synonymous				Exon 6	rs2298718	
JK*02N.09.210G_A_588A_G	JK*02	c.191G>A	p.Arg64Gln	1	<0.0001	<0.0001	Exon 4	rs114362217	
		c.210G>A	Synonymous				Exon 4	rs1796634758	
		c.588A>G	Synonymous				Exon 6	rs2298718	
JK*02N.17.588A_G	JK*02N.17	c.588A>G	Synonymous	859	0.0574	0.0456	Exon 6	rs2298718	
JK*02N.17.588A_G_957G_A	JK*02N.17	c.588A>G	Synonymous	1	<0.0001	0.0456	Exon 6	rs2298718	
		c.957G>A	Synonymous				Exon 9	rs376375507	
JK*02N.17.588A_G_1095T_C	JK*02N.17	c.588A>G	Synonymous	1	<0.0001	0.0039	Exon 6	rs2298718	
		c.1095T>C	Synonymous				Exon 10	rs28898897	
JK*02W.03.M7V	JK*02W.03	c.19A>G	p.Met7Val	1	<0.0001	<0.0001	Exon 3	rs138369087	
JK*02W.03.V10M_V76I_303G_A^||^	JK*02W.03	c.28G>A	p.Val10Met	4	<0.0001	0.0179	Exon 3	rs113578396	
		c.226G>A	p.Val76Ile				Exon 4	rs113029149	
		c.303G>A	Synonymous				Exon 4	rs28994287	
JK*02W.03.56irsA_549G_A	JK*02W.03	c.56insA	p.Asn20Lysfs*20	1	<0.0001	−	Exon 3	Novel	MW604799
		c.549G>A	Synonymous			0.0001	Exon 6	rs375370757	
JK*02W.03.69G_A	JK*02W.03	c.69G>A	Synonymous	1	<0.0001	0.0002	Exon 3	rs140137213	
JK*02W.03.Y37X	JK*02W.03	c.111T>G	p.Tyr37*	1	<0.0001	−	Exon 3	Novel	MW604800
JK*02W.03.L63F	JK*02W.03	c.187C>T	p.Leu63Phe	5	<0.0001	0.0001	Exon 4	rs201716471	
JK*02W.03.V76I^||^	JK*02W.03	c.226G>A	p.Val76Ile	1	<0.0001	0.022	Exon 4	rs113029149	
JK*02W.03.P90S	JK*02W.03	c.268C>T	p.Pro90Ser	18	0.0012	<0.0001	Exon 4	rs147790844	
JK*02W.03.T95I_V175I	JK*02W.03	c.284C>T	p.Thr95Ile	1	<0.0001	<0.0001	Exon 4	rs764618306	
		c.523G>A	p.Val 175Ile			<0.0001	Exon 6	rs138222201	
JK*02 W.03.D113E	JK*02W.03	c.339C>G	p.Asp1 13Glu	1	<0.0001	−	Exon 4	Novel	MW604791
JK*02 W.03.W144R	JK*02W.03	c.430T>C	p.Trp144Arg	1	<0.0001	−	Exon 5	Novel	MW604798
JK*02 W.03.M167V	JK*02W.03	c.499A>G	p.Met167Val	127	0.0058	0.0082	Exon 6	rs2298719	
JK*02W.03.582C_T	JK*02W.03	c.582C>T	Synonymous	15	0.0010	0.0035	Exon 6	rs34756616	
JK*02 W.03.597*C_T*	JK*02W.03	c.591C>T	Synonymous	1	<0.0001	<0.0001	Exon 6	rs748824511	
JK*02W.03.N27*1S*	JK*02W.03	c.632A>G	p.Asn211Ser	1	<0.0001	−	Exon 6	Novel	MW604795
JK*02W.03.667C_T	JK*02W.03	c.667C>T	Synonymous	1	<0.0001	<0.0001	Exon 7	rs748771470	
JK*02W.03.678A_T	JK*02W.03	c.678A>T	Synonymous	1	<0.0001	−	Exon 7	Novel	MW604788
JK*02W.03.840C_T	JK*02W.03	c.840C>T	Synonymous	3	<0.0001	−	Exon 8	Novel	MW604789
JK*02W.03.948C_G	JK*02W.03	c.948C>G	Synonymous	1	<0.0001	−	Exon 9	Novel	MW604790
JK*02W.03.957G_A	JK*02W.03	c.957G>A	Synonymous	2	<0.0001	<0.0001	Exon 9	rs376375507	
JK*02W.03.L322F	JK*02W.03	c.964C>T	p.Leu322Phe	1	<0.0001	−	Exon 9	Novel	MW604794
JK*02W.03.G325S	JK*02W.03	c.973G>A	p.Gly325Ser	1	<0.0001	0.0001	Exon 9	rs140320419	
JK*02W.03.1047C_T	JK*02W.03	c.1047C>T	Synonymous	1	<0.0001	−	Exon 10	Novel	MW604787
JK*02W.03.K355N_E9+9G_T	JK*02W.03	c.1065A>G	p.Lys355Asn	1	<0.0001	−0.0003	Exon 10	Novel	MW604793
		c.996+9G>T	Synonymous				Intron 9	rs188063122	
JK*02W.03.P363H	JK*02W.03	c.1088C>A	p.Pro363His	1	<0.0001	−	Exon 10	Novel	MW604796
JK*02W.03.1095T_C	JK*02W.03	c.1095T>C	Synonymous	176	0.0118	0.0039	Exon 10	rs28898897	
JK*02W.03.E371K	JK*02W.03	c.1111G>A	p.Glu371Lys	2	<0.0001	<0.0001	Exon 10	rs756254780	
JK*02 W.03.F376L	JK*02W.03	c.1126T>C	p.Phe376Leu	1	<0.0001	<0.0001	Exon 10	rs772420027	
JK*02W.03.I375V	JK*02W.03	c.1123A>G	p.Ile375Val	1	<0.0001	−	Exon 10	Novel	MW604792
JK*02W.03.V385M	JK*02W.03	c.1153G>A	p.Val385Met	1	<0.0001	−	Exon 10	Novel	MW604797
JK*02W.03.E8-38C_G	JK*02W.03	c.812-38C>G	−	1	<0.0001	<0.0001	Intron 7	rs369987138	
JK*02W.03.E9+9G_T	JK*02W.03	c.996+9G>T	−	1	<0.0001	0.0004	Intron 9	rs188063122	
JK*02W.04.588A_G	JK*02	c.130G>A	p.Glu44Lys	6	<0.0001	0.1376	Exon 3	rs2298720	
		c.588A>G	Synonymous				Exon 6	rs2298718	
JK*02W.04.M167V_588A_G	JK*02	c.130G>A	p.Glu44Lys	3	<0.0001	0.0082	Exon 3	rs2298720	
		c.499A>G	p.Met167Val				Exon 6	rs2298719	
		c.588A>G	Synonymous				Exon 6	rs2298718	

* Enumeration of nucleotide and amino acid changes according to reference allele JK*01.

§ SNV frequency as published by gnom AD database V3.1.1 [5].

† SNV ID obtained from gnomAD database.

|| Variants were referenced by ISBT to result in altered RBC phenotype.

**Table 5:** Frequencies of ISBT-referenced alleles and alleles that were detected at least five times in various blood donor panels

Allele name*	WEUR (*n* = 531) *n* (%)	TUR (*n* = 2,280) *n* (%)	SYR (*n* = 706) *n* (%)	SSAF (*n* = 70) *n* (%)	SEEA (*n*=157) *n* (%)	SAS (*n* = 238) *n* (%)	CSAM (*n*= 78) *n* (%)	NAF (*n* = 227) *n* (%)	IRAQ (*n* = 167) *n* (%)	IRAN (*n* = 328) *n* (%)	EEKCA (*n* = 849) *n* (%)	ARPE (*n* = 195) *n* (%)	OTH (*n* = 2,207) *n* (%)	Total (*n* = 8,033) *n* (%)
JK1 alleles (total)	540 (50.85)	2,347(51.47)	730 (53.29)	100 (71.43)	158(50.32)	273 (57.35)	82 (52.56)	274 (60.35)	160(47.91)	339(51.68)	868 (51.12)	208 (53.33)	2,282 (51.70)	8,360 (52.04)
JK2 alleles (total)	522 (49.15)	2,213 (48.53)	682 (46.71)	40 (28.57)	156(49.68)	203 (42.65)	74 (47.44)	180 (39.65)	174 (52.09)	317(48.32)	830 (48.88)	182 (46.67)	2,132 (48.30)	7,706 (47.96)
ISBT referenced alleles														
JK*01	428 (40.28)	1,721 (37.74)	561 (39.73)	48 (34.29)	49 (15.61)	149 (31.30)	54 (34.62)	184 (40.53)	120(35.93)	253 (38.57)	685 (40.34)	155 (39.74)	1,639(35.30)	6,034 (37.56)
JK*01W.01	2 (0.19)	14 (0.31)	1 (0.07)	−	−	1 (0.21)	−	−	1 (0.30)	1 (0.15)	7(0.41)	−	5 (0.11)	32 (0.20)
JK*01W.03	−	−	−	−	−	−	−	−	−	−	−	−	1 (0.02)	1 (0.006)
JK*01W.04	−	−	−	−	−	−	−	−	−	−	−	−	1 (0.02)	1 (0.006)
JK*01W.06	7 (0.66)	19(0.42)	7 (0.50)	9 (6.43)	−	1 (0.21)	6 (3.85)	9 (1.98)	−	4(0.61)	4 (0.24)	2 (0.51)	29 (0.66)	97 (0.60)
JK*01N.09	−	−	−	−	−	−	−	−	−	−	3 (0.18)	−	−	3 (0.024)
JK*01N.18	−	1 (0.02)	−	−	−	−	−	−	−	−	1 (0.06)	−	−	2 (0.012)
JK*01N.20	3 (0.29)	35 (0.77)	15 (1.06)	9 (6.43)	−	−	−	19(4.19)	5 (1.50)	1 (0.15)	8 (0.47)	4(1.03)	35 (0.80)	134 (0.84)
JK*02	−	−	−	−	−	1 (0.22)	−	−	−	−	−	−	−	1 (0.006)
JK*02W.03	425 (40.00)	1,832 (39.98)	561 (39.73)	37 (26.43)	91 (29.17)	171 (35.92)	68 (43.59)	162 (35.68)	139(41.62)	260 (39.63)	689 (40.58)	157(40.26)	1,765 (39.99)	6,357 (39.68)
JK*02N.17	−	−	−	−	−	−	−	−	−	−	−	−	1 (0.02)	1 (0.006)
Non referenced alleles														
JK*01W.01.588A_G	93 (9.25)	535 (11.75)	135 (9.56)	29(20.71)	104 (33.12)	119(25.00)	20(12.82)	58(12.78)	33 (9.88)	78 (11.89)	158(9.31)	44 (11.28)	560 (12.68)	1,966 (12.24)
JK*02N.17.588A_G	72 (6.79)	263 (5.77)	89 (6.30)	2 (1.43)	3 (0.96)	28 (5.88)	6 (3.85)	8(1.76)	24(7.19)	43 (6.55)	116(6.83)	14(3.59)	253 (5.73)	921 (5.73)
JK*02W.03.1095T_C	10(0.94)	62 (1.36)	20(1.42)	−	−	1 (0.21)	−	8(1.76)	4 (1.20)	6(0.91)	18(1.06)	8 (2.05)	52 (1.18)	189(1.18)
JK*02W.03.M167V	4 (0.38)	37(0.81)	4 (0.28)	−	52 (16.67)	−	−	−	−	1 (0.15)	1 (0.06)	−	36 (0.82)	135 (0.84)
JK*02W.03.P90S	1 (0.10)	4 (0.09)	−	−	−	1 (0.21)	−	−	5 (1.50)	4(0.61)	−	−	5 (0.11)	20(0.12)
JK*02W.03.582C**_***T*	6 (0.57)	−	1 (0.07)	−	2 (0.64)	−	−	1 (0.22)	−	−	1 (0.06)	−	5 (0.11)	16 (0.10)
JK*01.Q21R	1 (0.10)	2 (0.04)	2 (0.14)	−	−	−	−	−	−	−	1 (0.06)	−	2 (0.04)	8 (0.05)
JK*01.L148F	−	2 (0.04)	−	−	−	−	−	−	−	−	−	1 (0.26)	5 (0.11)	8 (0.05)
JK*01.E5-23G_A	−	2 (0.04)	2 (0.14)	−	−	−	−	1 (0.22)	1 (0.30)	−	−	−	2 (0.04)	8 (0.05)
JK*02W.04.588A_G	−	1 (0.02)	1 (0.07)	−	1 (0.32)	1 (0.21)	−	−	1 (0.32)	−	−	−	1 (0.02)	6 (0.04)
JK*01.1095T_C	−	5 (0.11)	1 (0.07)	−	−	−	−	−	−	−	−	−	−	6 (0.04)
JK*01W.02.402T_C_588A_G	2 (0.19)	−	−	2 (1.43)	−	−	−	−	−	−	−	−	2 (0.04)	6 (0.04)
JK*02W.03.L63F	−	1 (0.02)	2 (0.14)	−	−	−	−	−	−	−	−	−	2 (0.04)	5 (0.03)
JK*02N.01.588A_G	−	−	−	−	5 (1.60)	−	−	−	−	−	−	−	−	5 (0.03)
JK*02N.09.588A_G	−	2 (0.04)	−	−	−	−	−	−	−	−	1 (0.06)	−	2 (0.04)	6 (0.04)

* Details of alleles are given in Table 4.
