# Supplementary material for: High-Throughput Next-Generation Sequencing of the Kidd Blood Group: Unexpected Antigen Expression Properties of Four Alleles and Detection of Novel Variants
Source: Transfus Med Hemother. 2022 Jul 26;50(1):51–65. doi: 10.1159/000525326 (PMC9911998; doi:10.1159/000525326)
Supplement: Supplementary file 1 — Supplementary data [file tmh-0050-0051-s01.docx]

Supplementary Table S1. Blood group systems and HPA included in the NGS test system.

| **ISBT System No.** | **System Name** | **System Symbol** | **Gene(s)** |
| --- | --- | --- | --- |
| 001 | ABO | ABO | *ABO* |
| 002 | MNS | MNS | *GYPA*  *GYPB*  *GYPE* |
| 003 | P1PK | P1PK | *A4GALT* |
| 004 | Rh | RH | *RHCE*  *RHD* |
| 005 | Lutheran | LU | *B-CAM* |
| 006 | Kell | KEL | *KEL* |
| 007 | Lewis | LE | *FUT3* |
| 008 | Duffy | FY | *DARC* |
| 009 | Kidd | JK | *SLC14A1* |
| 010 | Diego | DI | *SLC4A1* |
| 011 | Cartwright | YT | *ACHE* |
| 014 | Dombrock | DO | *ART4* |
| 015 | Colton | CO | *AQP1* |
| 018 | H | H | *FUT1*  *FUT2* |
| 020 | Gerbich | GYPC | *GYPC* |
| 021 | Cromer | CROM | *CD55* |
| 022 | Knops | KN | *CR1* |
| 023 | Indian | IN | *CD44* |
| 032 | Junior | JR | *ABCG2* |
| 033 | Langereis | LAN | *ABCB6* |
| 034 | Vel | VEL | *SMIM1* |
| 036 | Augustine | AUG | *SLC29A1* |
|  | HPA-1 / HPA-4 |  | *ITGB3* |
|  | HPA-2 |  | *GP1BA* |
|  | HPA-3 |  | *ITGA2B* |
|  | HPA-5 |  | *ITGA2* |
|  | HPA-15 |  | *CD109* |
