# Supplementary material for: High-Throughput Next-Generation Sequencing of the Kidd Blood Group: Unexpected Antigen Expression Properties of Four Alleles and Detection of Novel Variants
Source: Transfus Med Hemother. 2022 Jul 26;50(1):51–65. doi: 10.1159/000525326 (PMC9911998; doi:10.1159/000525326)
Supplement: Supplementary file 2 — Supplementary data [file tmh-0050-0051-s02.docx]

Supplementary Table S2. Characteristics of primers and data metrics.

Depth of coverage of exon 3 to 10 amplicons of the *SLC14A1* gene obtained for validation sample set. Minimum acceptance of depth of coverage was set at 80 and 30 for homozygous and heterozygous fragments, respectively. ^*^Numbering is referenced to Genome build GRCh37.1 / hg19, chromosome 18.

| **Target** | **Amplified Region^*^** | **Amplicon Size (bp)** | **Depth of coverage for homozygous samples** | | | **Depth of coverage of coverage for heterozygous samples** | | |
| --- | --- | --- | --- | --- | --- | --- | --- | --- |
|  |  |  | **Maximum** | **Minimum** | **Mean** | **Maximum** | **Minimum** | **Mean** |
| Exon 3 | 43,310,204..43,310,640 | 437 | 3178 | 80 | 605.91 | 1409 | 61 | 321.59 |
| Exon 4 | 43,310,772..43,311,228 | 457 | 2378 | 81 | 597.45 | 1690 | 75 | 306.01 |
| Exon 5 | 43,314,188..43,314568 | 381 | 1821 | 80 | 300.6 | 1465 | 58 | 166.44 |
| Exon 6 | 43,316,359..43,316,727 | 369 | 4016 | 80 | 873.59 | 1296 | 55 | 450.69 |
| Exon 7 | 43,318,864..43,319,352 | 489 | 3661 | 80 | 690.75 | 1056 | 52 | 352.04 |
| Exon 8 | 43,319,333..43,319,793 | 461 | 1605 | 81 | 413.06 | 2047 | 56 | 216.78 |
| Exon 9 | 43,328,196..43,328,591 | 396 | 3682 | 82 | 616.34 | 1742 | 59 | 340.31 |
| Exon 10 | 43,329,608..43,329,971 | 364 | 3875 | 80 | 843.48 | 838 | 51 | 440.02 |
