# Supplementary material for: High-Throughput Next-Generation Sequencing of the Kidd Blood Group: Unexpected Antigen Expression Properties of Four Alleles and Detection of Novel Variants
Source: Transfus Med Hemother. 2022 Jul 26;50(1):51–65. doi: 10.1159/000525326 (PMC9911998; doi:10.1159/000525326)
Supplement: Supplementary file 3 — Supplementary data [file tmh-0050-0051-s03.docx]

Supplementary Table S3. Depth of coverage of exon 3 to 10 amplicons of the *SLC14A1* gene obtained for 95 repeated samples. Minimum acceptance of depth of coverage was set at 80 and 30 for homozygous and heterozygous fragments, respectively.

|  |  | **Depth of coverage for  homozygous amplicons** | | | **Depth of coverage of heterozygous amplicons** | | |
| --- | --- | --- | --- | --- | --- | --- | --- |
| **Run number** | **Amplicon** | **Minimum** | **Maximum** | **Mean** | **Minimum** | **Maximum** | **Mean** |
| 1 | Exon 3 | 92 | 635 | 300.76 | 76 | 269 | 152.60 |
| 2 |  | 90 | 3159 | 492.93 | 131 | 552 | 295.59 |
| 3 |  | 128 | 3012 | 958.78 | 128 | 1465 | 350.13 |
| 1 | Exon 4 | 192 | 945 | 451.73 | 93 | 514 | 232.40 |
| 2 |  | 101 | 1112 | 493.32 | 110 | 599 | 304.70 |
| 3 |  | 85 | 1261 | 394.56 | 55 | 792 | 198.60 |
| 1 | Exon 5 | 80 | 231 | 121.83 | 61 | 125 | 85.77 |
| 2 |  | 82 | 584 | 212.16 | 61 | 331 | 148.39 |
| 3 |  | 83 | 1327 | 352.08 | 63 | 617 | 278.34 |
| 1 | Exon 6 | 135 | 687 | 343.67 | 64 | 388 | 177.96 |
| 2 |  | 105 | 2265 | 884.83 | 89 | 1063 | 510.04 |
| 3 |  | 149 | 2318 | 698.53 | 118 | 1047 | 460.22 |
| 1 | Exon 7 | 168 | 868 | 455.98 | 102 | 744 | 219.72 |
| 2 |  | 105 | 1767 | 384.26 | 69 | 521 | 208.90 |
| 3 |  | 95 | 3661 | 1122.08 | 129 | 1680 | 369.64 |
| 1 | Exon 8 | 95 | 565 | 250.47 | 71 | 306 | 138.13 |
| 2 |  | 81 | 780 | 368.73 | 79 | 463 | 219.04 |
| 3 |  | 86 | 1011 | 299.33 | 59 | 500 | 289.28 |
| 1 | Exon 9 | 86 | 419 | 241.23 | 55 | 279 | 124.99 |
| 2 |  | 119 | 1677 | 433.05 | 106 | 583 | 241.41 |
| 3 |  | 129 | 2673 | 849.12 | 106 | 1356 | 214.47 |
| 1 | Exon 10 | 136 | 1497 | 613.20 | 146 | 770 | 361.38 |
| 2 |  | 113 | 1970 | 1026.54 | 340 | 1013 | 604.72 |
| 3 |  | 122 | 2095 | 660.15 | 99 | 948 | 423.69 |
