# Supplementary material for: High-Throughput Next-Generation Sequencing of the Kidd Blood Group: Unexpected Antigen Expression Properties of Four Alleles and Detection of Novel Variants
Source: Transfus Med Hemother. 2022 Jul 26;50(1):51–65. doi: 10.1159/000525326 (PMC9911998; doi:10.1159/000525326)
Supplement: Supplementary file 4 — Supplementary data [file tmh-0050-0051-s04.docx]

Supplementary Table S4. Discrepant results. Serological RBC phenotyping results did not match genotyping data of five blood donor samples. Details of alleles are given in Table 3.

| **NGS genotype** | **Deduced RBC phenotype** | **Serological RBC phenotype** |
| --- | --- | --- |
| JK*01/01 | JK:1 | JK:1,2 |
| JK*01/02W.03 | JK:1,2^weak^ | JK:2 |
| JK*01/02W.03.M167V | JK:1,2^weak^ | JK:2 |
| JK*01/02N.17.588A_G | JK:1 | JK:2 |
| JK*02W.03/02N.17.588A_G | JK:2^weak^ | JK:1,2 |
