# Supplementary material for: High-Throughput Next-Generation Sequencing of the Kidd Blood Group: Unexpected Antigen Expression Properties of Four Alleles and Detection of Novel Variants
Source: Transfus Med Hemother. 2022 Jul 26;50(1):51–65. doi: 10.1159/000525326 (PMC9911998; doi:10.1159/000525326)
Supplement: Supplementary file 5 — Supplementary data [file tmh-0050-0051-s05.docx]

Supplementary Table S5. Genotyping data of 8,033 blood donor samples with different countries of origin.

| **Kidd Genotype*** | **WEUR (n=531),  n(%)** | **TUR (n=2,280), n(%)** | **SYR (n=706), n(%)** | **SSAF (n=70), n(%)** | **SEEA (n=157), n(%)** | **SAS (n=238), n(%)** | **CSAM (n=78), n(%)** | **NAF (n=227), n(%)** | **IRAQ (n=167), n(%)** | **IRAN (n=328), n%** | **EEKCA (n=849), n(%)** | **ARPE (n=195), n(%)** | **OTH (n=2,207), n(%)** | **Number of samples (n=8,033) n(%)** |
| --- | --- | --- | --- | --- | --- | --- | --- | --- | --- | --- | --- | --- | --- | --- |
| JK1/JK1 (Total) | 135 (25.47) | 615 (26.97) | 196 (27.76) | 34 (48.57) | 42 (26.92) | 79 (33.19) | 22 (28.20) | 83 (36.56) | 32 (19.16) | 77 (23.48) | 223 (26.27) | 61 (31.28) | 506 (22.93) | 2,171  (27.03) |
| JK1/JK2 (Total) | 271 (51.13) | 1,117 (48.99) | 338 (47.88) | 32 (45.71) | 72 (45.86) | 115 (48.32) | 38 (48.72) | 108 (47.57) | 96 (57.49) | 185 (56.40) | 424 (49.94) | 87 (44.62) | 1,121 (50.79) | 4,003  (49.83) |
| JK2/JK2 (Total) | 125 (23.58) | 548 (24.04) | 172 (24.36) | 4 (5.71) | 43 (27.39) | 44 (18.49) | 18 (23.08) | 36 (15.86) | 39 (23.53) | 66 (20.12) | 203 (23.91) | 46 (23.59) | 580 (26.28) | 1,859  (23.14) |
| *JK*01/01* | 84  (15.82) | 329  (14.43) | 108  (15.30) | 6 (8.57) | 3  (1.92) | 17 (7.14) | 9  (11.54) | 35  (15.42) | 16  (9.58) | 44  (13.41) | 128  (15.08) | 36  (18.46) | 316  (14.32) | 1,131  (14.08) |
| *JK*01/01.105T_C* | - | - | 1 (0.14) | - | - | - | - | - | - | - | - | - | - | 1 (0.01) |
| *JK*01/01.1095T_C* | - | - | 1 (0.14) | - | - | - | - | - | - | - | - | - | - | 1 (0.01) |
| *JK*01/01.159C_T* | 1 (0.19) | - | - | - | - | - | - | - | - | - | - | - | - | 1 (0.01) |
| *JK*01/01.163delG* | - | 1 (0.04) | - | - | - | - | - | - | - | - | - | - | - | 1 (0.01) |
| *JK*01/01.E5+12T_C* | - | - | - | - | - | - | - | - | - | - | - | 1 (0.52) | - | 1 (0.01) |
| *JK*01/01.E5-19T_A* | - | - | 1 (0.14) | - | - | - | - | - | - | - | - | - | - | 1 (0.01) |
| *JK*01/01.E5-23G_A* | - | 2 (0.09) | 1 (0.14) | - | - | - | - | 1 (0.44) | - | - | - | - | - | 4 (0.05) |
| *JK*01/01.E8+31G_A* | - | - | - | 1 (1.43) | - | - | - | - | - | - | - | - | - | 1 (0.01) |
| *JK*01/01.E9+18T_C* | - | - | 1 (0.14) | - | - | - | - | - | - | - | - | - | - | 1 (0.01) |
| *JK*01/01.L148F* | - | - | - | - | - | - | - | - | - | - | - | 1 (0.52) | 3 (0.13) | 4 (0.05) |
| *JK*01/01.L364F* | - | 1 (0.04) | 1 (0.14) | - | - | - | - | - | - | - | - | - | 1 (0.04) | 3 (0.04) |
| *JK*01/01.M352I* | - | - | - | - | - | - | - | - | - | 1 (0.30) | - | - | - | 1 (0.01) |
| *JK*01/01.N373I* | - | - | - | - | - | - | - | - | - | - | - | - | 1 (0.04) | 1 (0.01) |
| *JK*01/01.Q21R* | 1 (0.19) | 2 (0.09) | 2 (0.28) | - | - | - | - | - | - | - | 1 (0.12) | - | - | 6 (0.08) |
| *JK*01/01.R64Q* | - | - | - | - | - | - | - | - | - | - | 1 (0.12) | - | - | 1 (0.01) |
| *JK*01/01.V367A* | - | - | - | - | - | - | - | - | - | - | - | 1 (0.52) | - | 1 (0.01) |
| *JK*01/01.V54M* | - | - | - | - | - | 1 (0.42) | - | - | - | - | - | - | - | 1 (0.01) |
| *JK*01/01.V8F* | - | 1 (0.04) | - | - | - | - | - | - | - | - | - | - | - | 1 (0.01) |
| *JK*01/01N.09.V175I* | - | - | - | 1 (1.43) | - | - | - | - | - | - | - | - | - | 1 (0.01) |
| *JK*01/01N.18* | - | - | - | - | - | - | - | - | - | - | 1 (0.12) | - | - | 1 (0.01) |
| *JK*01/01N.20* | 2 (0.38) | 16 (0.70) | 11 (1.56) | 3 (4.29) | - | - | - | 6 (2.64) | 1 (0.60) | - | 1 (0.12) | - | 12 (0.54) | 52 (0.65) |
| *JK*01/01W.01* | 1 (0.19) | 7 (0.31) | 1 (0.14) | - | - | 1 (0.42) | - | - | - | 1 (0.30) | 4 (0.47) | - | 4 (0.18) | 19 (0.22) |
| *JK*01/01W.01.516C_T_588A_G* | - | 1 (0.04) | - | - | - | - | - | - | - | - | - | - | - | 1 (0.01) |
| *JK*01/01W.01.588A_G* | 32  (6.03) | 199 (8.73) | 50 (7.08) | 8  (11.43) | 18  (11.46) | 45  (18.91) | 7 (8.97) | 22 (9.69) | 12 (7.19) | 23 (7.01) | 69  (8.13) | 17 (8.76) | 168  (7.76) | 670 (8.34) |
| *JK*01/01W.01.D11N_588A_G* | - | - | - | - | - | - | - | - | - | - | - | - | 1 (0.04) | 1 (0.01) |
| *JK*01/01W.01.P205S_588A_G* | - | - | - | - | 1 (0.64) | - | - | - | - | - | - | - | - | 1 (0.01) |
| *JK*01/01W.02.402T_C_588A_G* | 1 (0.19) | - | - | 1 (1.43) | - | - | - | - | - | - | - | - | 2 (0.09) | 4 (0.05) |
| *JK*01/01W.04.303G_A* | 1 (0.19) | - | 1 (0.14) | - | - | - | - | - | - | - | - | - | - | 2 (0.02) |
| *JK*01/01W.06* | 1 (0.19) | 10 (0.44) | 3 (0.42) | 4 (5.71) | - | 1 (0.42) | 3 (3.85) | 5 (2.20) | - | 1 (0.30) | 3 (0.35) | 1 (0.52) | 11 (0.50) | 43 (0.54) |
| ***JK*01/02N.05.588A_G^§^*** | - | - | - | - | - | - | - | - | - | - | - | - | 1 (0.04) | 1 (0.01) |
| ***JK*01/02N.09.588A_G^§^*** | - | - | - | - | - | - | - | - | - | - | 1 (0.12) | - | 2 (0.09) | 3 (0.04) |
| *JK*01/02N.17* | - | - | - | - | - | - | - | - | - | - | - | - | 1 (0.04) | 1 (0.01) |
| *JK*01/02N.17.588A_G* | 31  (5.85) | 108 (4.74) | 33 (4.67) | 1 (1.43) | 1 (0.64) | 9 (3.78) | 2 (2.56) | 3 (1.32) | 11 (6.59) | 24 (7.32) | 47 (5.54) | 2 (1.03) | 89 (4.03) | 361 (4.51) |
| *JK*01/02W.03* | 181  (34.15) | 664  (29.12) | 227  (32.15) | 16  (22.86) | 17  (10.90) | 58  (24.37) | 24  (30.77) | 72  (31.72) | 60  (35.93) | 109  (34.04) | 289  (33.94) | 57  (29.38) | 667  (30.22) | 2,441 (30.4) |
| *JK*01/02W.03.1047C_T* | - | - | - | - | - | - | - | - | - | - | - | - | 1 (0.04) | 1 (0.01) |
| *JK*01/02W.03.1095T_C* | 4 (0.75) | 25 (1.10) | 5 (0.71) | - | - | - | - | 5 (2.20) | 2 (1.20) | 2 (0.61) | 10 (1.18) | 2 (1.03) | 17 (0.63) | 72 (0.90) |
| *JK*01/02W.03.582C_T* | 3 (0.58) | - | - | - | - | - | - | - | - | - | - | - | 4 (0.17) | 7 (0.09) |
| *JK*01/02W.03.E371K* | - | - | - | - | - | - | - | - | - | - | 2 (0.24) | - | - | 2 (0.02) |
| *JK*01/02W.03.F376L* | - | 1 (0.04) | - | - | - | - | - | - | - | - | - | - | - | 1 (0.01) |
| *JK*01/02W.03.G325S* | - | - | 1 (0.14) | - | - | - | - | - | - | 1 (0.30) | - | - | - | 2 (0.02) |
| *JK*01/02W.03.I375V* | - | - | - | - | - | - | - | - | - | - | - | 1 (0.52) | - | 1 (0.01) |
| *JK*01/02W.03.L63F* | - | - | 1 (0.14) | - | - | - | - | - | - | - | - | - | - | 1 (0.01) |
| *JK*01/02W.03.M167V* | 1 (0.19) | 18 (0.79) | 4 (0.57) | - | 6 (3.85) | - | - | - | - | - | - | - | 9 (0.39) | 38 (0.47) |
| *JK*01/02W.03.M7V* | - | 1 (0.04) | - | - | - | - | - | - | - | - | - | - | - | 1 (0.01) |
| *JK*01/02W.03.P90S* | - | 2 (0.09) | - | - | - | - | - | - | 2 (1.20) | 3 (0.91) | - | - | 1 (0.04) | 8 (0.10) |
| *JK*01/02W.03.V10M_V76I_303G_A* | - | 1 (0.04) | - | 1 (1.43) | - | - | - | - | - | - | - | - | - | 2 (0.02) |
| *JK*01/02W.03.W144R* | - | 1 (0.04) | - | - | - | - | - | - | - | - | - | - | - | 1 (0.01) |
| ***JK*01/02W.04.M167V_588A_G^$^*** | - | 2 (0.09) | - | - | - | - | - | - | - | - | - | - | 1 (0.04) | 3 (0.04) |
| *JK*01.105T_C/02W.03* | - | 1 (0.04) | - | - | - | - | - | - | - | - | - | - | - | 1 (0.01) |
| *JK*01.1095T_C/01W.01.588A_G* | - | 1 (0.04) | - | - | - | - | - | - | - | - | - | - | - | 1 (0.01) |
| *JK*01.1095T_C/02W.03* |  | 4 (0.18) |  |  |  |  |  |  |  |  |  |  |  | 4 (0.05) |
| *JK*01.159C_T/02W.03* | 1 (0.19) | - | - | - | - | - | - | - | - | - | - | - | - | 1 (0.01) |
| *JK*01.E4+10C_T/01W.01.588A_G* | - | 2 (0.09) | - | - | - | - | - | - | - | - | - | - | - | 2 (0.02) |
| *JK*01.E5+12T_C/01W.06* | - | - | - | - | - | - | 1 (1.28) | - | - | - | - | - | - | 1 (0.01) |
| *JK*01.E5-23G_A/02N.17.588A_G* | - | - | - | - | - | - | - | - | - | - | - | - | 1 (0.04) | 1 (0.01) |
| *JK*01.E5-23G_A/02W.03* | - | - | 1 (0.14) | - | - | - | - | - | 1 (0.60) | - | - | - | 1 (0.04) | 3 (0.04) |
| *JK*01.E9-13T_C/02W.03* | - | - | - | - | - | - | - | - | - | - | - | - | 1 (0.04) | 1 (0.01) |
| *JK*01.G27R/02W.03* | - | - | - | - | - | 1 (0.42) | - | - | - | - | - | - | - | 1 (0.01) |
| *JK*01.G96V/02W.03* | - | - | - | - | - | - | - | - | - | - | - | - | 1 (0.04) | 1 (0.01) |
| *JK*01.L148F/02N.17.588A_G* | - | 1 (0.04) | - | - | - | - | - | - | - | - | - | - | 1 (0.04) | 2 (0.02) |
| *JK*01.L148F/02W.03* | - | 1 (0.04) | - | - | - | - | - | - | - | - | - | - | 1 (0.04) | 2 (0.02) |
| *JK*01.L188F_561C_T/02N.17.588A_G* | 1 (0.19) | - | - | - | - | - | - | - | - | - | - | - | - | 1 (0.01) |
| *JK*01.M352I/01W.01.588A_G* | - | 2 (0.09) | - | - | - | - | - | - | - | - | - | - | - | 2 (0.02) |
| *JK*01.M352I/02W.03* | - | 1 (0.04) | - | - | - | - | - | - | - | - | - | - | - | 1 (0.01) |
| *JK*01.Q21R/01W.01.588A_G* | - | - | - | - | - | - | - | - | - | - | - | - | 1 (0.04) | 1 (0.01) |
| *JK*01.Q21R/02W.03* |  |  |  |  |  |  |  |  |  |  |  |  | 1 (0.04) | 1 (0.01) |
| *JK*01.T308I/02W.03* | - | - | - | - | - | - | - | - | - | - | - | - | 1 (0.04) | 1 (0.01) |
| *JK*01.T346M/01W.01.588A_G* | - | - | - | - | - | - | - | - | - | - | - | - | 1 (0.04) | 1 (0.01) |
| *JK*01.V76A/01N.20* | - | - | - | - | - | - | - | - | - | - | - | - | 1 (0.04) | 1 (0.01) |
| *JK*01.V87I/02W.03* | - | - | 1 (0.14) | - | - | - | - | - | - | - | - | - | - | 1 (0.01) |
| ***JK*01N.09/02W.03^§^*** | - | - | - | - | - | - | - | - | - | - | 3 (0.35) | - | - | 3 (0.04) |
| ***JK*01N.18/02W.03^§^*** | - | 1 (0.04) | - | - | - | - | - | - | - | - | - | - | - | 1 (0.01) |
| *JK*01N.20/01N.20* | - | - | 1 (0.14) | - | - | - | - | 1 (0.44) | - | - | - | - | - | 2 (0.02) |
| *JK*01N.20/01W.06.V54M* | - | - | - | - | - | - | - | 1 (0.44) | - | - | - | - | - | 1 (0.01) |
| *JK*01N.20/02N.17.588A_G* | - | 1 (0.04) | - | - | - | - | - | 1 (0.44) | - | - | 1 (0.12) | - | 2 (0.09) | 5 (0.06) |
| *JK*01N.20/02W.03* | - | 14 (0.61) | 1 (0.14) | 2 (2.6) | - | - | - | 6 (2.64) | 3 (1.80) | 1 (0.30) | 4 (0.47) | 4 (2.06) | 12 (0.54) | 47 (0.59) |
| ***JK*01N.20/02W.04.588A_G^$^*** | - | - | - | - | - | - | - | - | 1 (0.60) | - | - | - | - | 1 (0.01) |
| *JK*01W.01/01N.20* | - | 2 (0.09) | - | - | - | - | - | - | - | - | - | - | - | 2 (0.02) |
| *JK*01W.01/01W.01.588A_G* | 1 (0.19) | - | - | - | - | - | - | - | - | - | 1 (0.12) | - | 1 (0.04) | 3 (0.04) |
| *JK*01W.01/02N.17.588A_G* | - | 5 (0.22) | - | - | - | - | - | - | - | - | 2 (0.24) | - | - | 7 (0.09) |
| *JK*01W.01/02W.03* | - | - | - | - | - | - | - | - | 1 (0.60) | - | - | - | - | 1 (0.01) |
| *JK*01W.01.219C_T_588A_G/02W.03.M167V* | - | - | - | - | - | - | - | - | - | - | - | - | 1 (0.04) | 1 (0.01) |
| *JK*01W.01.279T_C_588A_G/01W.01.588A_G* | - | - | - | - | 1 (0.64) | - | - | - | - | - | - | - | - | 1 (0.01) |
| *JK*01W.01.516C_T_588A_G/02N.17.588A_G* | - | 1 (0.04) | - | - | - | - | - | - | - | - | - | - | - | 1 (0.01) |
| *JK*01W.01.588A_G/01N.20* | 1 (0.19) | 2 (0.09) | 1 (0.14) | 4 (5.88) | - | - | - | 2 (0.89) | - | - | 2 (0.24) | - | 8 (0.34) | 20 (0.25) |
| *JK*01W.01.588A_G/01W.01.588A_G* | 5 (0.94) | 36 (1.58) | 11 (1.56) | 2 (2.86) | 19 (12.18) | 13 (5.46) | 2 (2.56) | 8 (3.52) | 3 (1.80) | 6 (1.83) | 11 (1.30) | 4 (2.06) | 44 (1.94) | 164 (2.04) |
| *JK*01W.01.588A_G/01W.01.588A_G_E9-7T_C* | - | - | - | - | 1 (0.64) | - | - | - | - | - | - | - | - | 1 (0.01) |
| *JK*01W.01.588A_G/01W.01.F329V_588A_G* | - | - | - | - | - | 1 (0.42) | - | - | - | - | - | - | - | 1 (0.01) |
| *JK*01W.01.588A_G/01W.06* | 3 (0.58) | 1 (0.04) | - | 3 (4.29) | - | - | - | 1 (0.44) | - | 1 (0.30) | - | - | 3 (0.13) | 12 (0.15) |
| ***JK*01W.01.588A_G/02N.01.588A_G^§^*** | - | - | - | - | 1 (0.64) | - | - | - | - | - | - | - | - | 1 (0.01) |
| ***JK*01W.01.588A_G/02N.06.588A_G^§^*** | - | - | - | - | 1 (0.64) | - | - | - | - | - | - | - | - | 1 (0.01) |
| *JK*01W.01.588A_G/02N.17.588A_G* | 7 (1.32) | 19 (0.83) | 13 (1.84) | - | 1 (0.64) | 7 (2.94) | - | 1 (0.44) | 2 (1.20) | 6 (1.83) | 10 (1.30) | 2 (1.03) | 25 (1.08) | 94 (1.17) |
| *JK*01W.01.588A_G/02N.17.588A_G_957G_A* | - | - | - | - | - | - | - | - | - | - | - | - | 1 (0.04) | 1 (0.01) |
| *JK*01W.01.588A_G/02W.03* | 37  (6.98) | 225  (9.87) | 49 (6.94) | 10  (14.29) | 27  (17.31) | 34 (10.37) | 9  (11.54) | 14  (6.17) | 12 (7.19) | 34  (10.37) | 52 (6.12) | 18 (8.76) | 247  (11.19) | 771 (9.60) |
| *JK*01W.01.588A_G/02W.03.1095T_C* | - | 8 (0.35) | - | - | - | 1 (0.42) | - | 1 (0.44) | - | - | - | - | 10 (0.43) | 20 (0.25) |
| ***JK*01W.01.588A_G/02W.03.56insA_549G_A^§^*** | - | - | - | - | - | - | - | - | - | - | - | - | 1 (0.04) | 1 (0.01) |
| *JK*01W.01.588A_G/02W.03.582C_T* | - | - | - | - | 1 (0.64) | - | - | - | - | - | 1 (0.12) | - | - | 2 (0.02) |
| *JK*01W.01.588A_G/02W.03.591C_T* | - | - | - | - | 1 (0.64) | - | - | - | - | - | - | - | - | 1 (0.01) |
| *JK*01W.01.588A_G/02W.03.667C_T* | - | - | - | - | - | - | - | - | - | - | - | - | 1 (0.04) | 1 (0.01) |
| *JK*01W.01.588A_G/ 02W.03.K355N_E9+9G_T* | - | - | - | - | - | - | - | 1 (0.44) | - | - | - | - | - | 1 (0.01) |
| *JK*01W.01.588A_G/02W.03.L63F* | - | 1 (0.04) | - | - | - | - | - | - | - | - | - | - | - | 1 (0.01) |
| *JK*01W.01.588A_G/02W.03.M167V* | 1 (0.19) | 3 (0.13) | - | - | 14 (8.92) | - | - | - | - | - | - | - | 5 (0.22) | 23 (0.29) |
| *JK*01W.01.588A_G/02W.03.P90S* | 1 (0.19) | - | - | - | - | 1 (0.42) | - | - | 1 (0.60) | 1 (0.30) | - | - | - | 4 (0.05) |
| *JK*01W.01.588A_G/02W.03.T95I_V175I* | - | - | - | - | - | - | - | - | - | 1 (0.30) | - | - | - | 1 (0.01) |
| *JK*01W.01.D11N_588A_G/02N.17.588A_G* | - | - | - | - | - | - | - | - | - | 1 (0.30) | - | - | - | 1 (0.01) |
| *JK*01W.01.D11N_588A_G/02W.03* | 1 (0.19) | - | - | - | - | - | - | - | - | - | - | - | - | 1 (0.01) |
| *JK*01W.01.F329V_588A_G/01W.01.588A_G* | - | - | - | - | - | 1 (0.42) | - | - | - | - | - | - | - | 1 (0.01) |
| *JK*01W.01.T127I_588A_G/02W.03* | - | - | - | - | - | - | - | - | - | - | - | - | 1 (0.04) | 1 (0.01) |
| *JK*01W.02.402T_C_588A_G/01W.06* | - | - | - | - | - | - | - | - | - | - | - | - | 1 (0.04) | 1 (0.01) |
| ***JK*01W.02.402T_C_588A_G/02W.03^$^*** | - | - | - | 1 (1.43) | - | - | - | - | - | - | - | - | - | 1 (0.01) |
| ***JK*01W.02.402T_C_588A_G_E5-24C_T/02N.17.588A_G^$^*** | - | - | - | - | - | - | - | 1 (0.44) | - | - | - | - | - | 1 (0.01) |
| *JK*01W.03/01W.04.303G_A* | - | - | - | - | - | - | - | - | - | - | - | - | 1 (0.04) | 1 (0.01) |
| ***JK*01W.04/02W.03^§^*** | - | - | - | - | - | - | - | - | - | - | - | - | 1 (0.04) | 1 (0.01) |
| *JK*01W.04.303G_A/01W.06* | - | - | - | 1 (1.43) | - | - | - | - | - | - | - | - | 1 (0.04) | 2 (0.02) |
| *JK*01W.06/01N.20* | - | - | - | - | - | - | - | 1 (0.44) | - | - | - | - | - | 1 (0.01) |
| *JK*01W.06/01W.06* | - | - | 1 (0.14) | - | - | - | - | - | - | - | - | - | 1 (0.04) | 2 (0.02) |
| *JK*01W.06/02N.17.588A_G* | - | 1 (0.04) | 1 (0.14) | - | - | - | 1 (1.28) | - | - | - | - | - | 3 (0.13) | 6 (0.07) |
| *JK*01W.06/02W.03* | 2 (0.38) | 7 (0.31) | 1 (0.14) | 1 (1.43) | - | - | 1 (1.28) | 1 (0.44) | - | 2 (0.60) | 1 (0.12) | - | 8 (0.34) | 24 (0.30) |
| *JK*01W.06/02W.03.1095T_C* | - | - | - | - | - | - | - | 1 (0.44) | - | - | - | 1 (0.52) | 1 (0.04) | 3 (0.04) |
| *JK*01W.06/02W.03.V10M_V76I_303G_A* | - | - | - | - | - | - | - | - | - | - | - | - | 1 (0.04) | 1 (0.01) |
| *JK*01W.06.M167V/02N.17.588A_G* | - | - | - | - | - | - | 1 (1.28) | - | - | - | - | - | - | 1 (0.01) |
| *JK*01W.06.M167V/02W.03.M167V* | - | - | - | - | 2 (1.28) | - | - | - | - | - | - | - | - | 2 (0.02) |
| *JK*01W.06.V54M/02W.03* | - | - | - | - | - | - | - | 1 (0.44) | - | - | - | - | - | 1 (0.01) |
| *JK*02/02W.03* | - | - | - | - | - | 1 (0.42) | - | - | - | - | - | - | - | 1 (0.01) |
| ***JK*02N.01.588A_G/02N.01.588A_G^§^*** | - | - | - | - | 1 (0.64) | - | - | - | - | - | - | - | - | 1 (0.01) |
| ***JK*02N.09.588A_G/02N.09.588A_G^§^*** | - | 1 (0.04) | - | - | - | - | - | - | - | - | - | - | - | 1 (0.01) |
| *JK*02N.17.588A_G/02N.17.588A_G* | 1 (0.19) | 9 (0.39) | 2 (0.28) | - | - | - | - | - | 2 (1.20) | 1 (0.30) | 5 (0.59) | 2 (1.03) | 13 (0.14) | 35 (0.42) |
| *JK*02N.17.588A_G/02W.03.1095T_C* | 1 (0.19) | 1 (0.04) | 1 (0.14) | - | - | - | - | - | - | - | 3 (0.35) | 2 (1.03) | 2 (0.08) | 10 (0.12) |
| *JK*02N.17.588A_G/02W.03.840C_T* | - | 1 (0.04) | - | - | - | - | - | - | - | - | - | - | - | 1 (0.01) |
| *JK*02N.17.588A_G/02W.03.E9+9G_T* | 1 (0.19) | - | - | - | - | - | - | - | - | - | - | - | - | 1 (0.01) |
| *JK*02N.17.588A_G/02W.03.M167V* | - | 4 (0.18) | - | - | 1 (0.64) | - | - | - | - | - | - | - | 2 (0.08) | 7 (0.09) |
| *JK*02N.17.588A_G/02W.03.P363H* | - | - | - | - | - | - | - | - | 1 (0.60) | - | - | - | - | 1 (0.01) |
| *JK*02N.17.588A_G/02W.03.V385M* | - | - | - | - | - | - | - | - | - | 1 (0.30) | - | - | - | 1 (0.01) |
| *JK*02N.17.588A_G/02W.03.V76I* | 1 (0.19) | - | - | - | - | - | - | - | - | - | - | - | - | 1 (0.01) |
| *JK*02N.17.588A_G/02W.04.588A_G* | - | 1 (0.04) | - | - | - | - | - | - | - | - | - | - | - | 1 (0.01) |
| *JK*02N.17.588A_G_1095T_C/ 02W.03.V10M_V76I_303G_A* | - | - | 1 (0.14) | - | - | - | - | - | - | - | - | - | - | 1 (0.01) |
| *JK*02W.03/02N.01.588A_G* | - | - | - | - | 2 (2.94) | - | - | - | - | - | - | - | - | 2 (0.02) |
| *JK*02W.03 /02N.06.588A_G* | - | - | - | - | 1 (0.64) | - | - | - | - | - | - | - | - | 1 (0.01) |
| *JK*02W.03/02N.08.588A_G* | - | - | - | - | - | 1 (0.42) | - | - | - | - | - | - | 1 (0.04) | 2 (0.02) |
| *JK*02W.03/02N.09.210G_A_588A_G* | - | - | - | - | - | - | - | - | - | - | - | - | 1 (0.04) | 1 (0.01) |
| *JK*02W.03/02N.09.588A_G* | - | 1 (0.04) | - | - | - | - | - | - | - | - | - | - | - | 1 (0.01) |
| *JK*02W.03/02N.17.588A_G* | 28  (5.28) | 102 (4.47) | 37 (5.24) | 1 (1.43) | - | 12  (5.04) | 2 (2.56) | 2 (0.88) | 6 (3.59) | 9 (2.74) | 42  (4.95) | 4 (2.06) | 102  (4.62) | 347 (4.32) |
| *JK*02W.03/02W.03* | 82  (15.47) | 382  (16.75) | 113  (16.01) | 3 (4.29) | 14  (8.92) | 29  (12.18) | 16  (20.51) | 32  (14.10) | 26  (15.57) | 50  (15.24) | 145  (17.08) | 35  (18.04) | 335  (15.18) | 1,262 (15.71) |
| *JK*02W.03/02W.03.1095T_C* | 5 (0.94) | 28 (1.23) | 14 (1.98) | - | - | - | - | 1 (0.44) | 2 (1.20) | 4 (1.22) | 5 (0.59) | 3 (1.55) | 22 (1.00) | 84 (1.05) |
| *JK*02W.03/02W.03.582C_T* | 2 (0.38) | - | 1 (0.14) | - | 1 (0.64) | - | - | 1 (0.44) | - | - | - | - | 1 (0.04) | 6 (0.08) |
| *JK*02W.03/02W.03.678A_T* | - | 1 (0.04) | - | - | - | - | - | - | - | - | - | - | - | 1 (0.01) |
| *JK*02W.03/02W.03.69G_A* | 1 (0.19) | - | - | - | - | - | - | - | - | - | - | - | - | 1 (0.01) |
| *JK*02W.03/02W.03.840C_T* | - | 2 (0.09) | - | - | - | - | - | - | - | - | - | - | - | 2 (0.02) |
| *JK*02W.03/02W.03.948C_G* | - | - | - | - | - | - | - | - | - | - | - | 1 (0.52) | - | 1 (0.01) |
| *JK*02W.03/02W.03.957G_A* | - | - | - | - | - | - | - | - | - | - | - | 1 (0.52) | 1 (0.04) | 2 (0.02) |
| *JK*02W.03/02W.03.D113E* | - | - | - | - | - | - | - | - | - | - | - | - | 1 (0.04) | 1 (0.01) |
| *JK*02W.03/02W.03.E8-38C_G* | - | - | 1 (0.14) | - | - | - | - | - | - | - | - | - | - | 1 (0.01) |
| *JK*02W.03/02W.03.L322F* | - | - | - | - | - | - | - | - | - | - | 1 (0.12) | - | - | 1 (0.01) |
| *JK*02W.03/02W.03.L63F* | - | - | 1 (0.14) | - | - | - | - | - | - | - | - | - | 2 (0.09) | 3 (0.04) |
| *JK*02W.03/02W.03.M167V* | 2 (0.38) | 12 (0.53) | - | - | 16 (10.26) | - | - | - | - | 1 (0.30) | 1 (0.12) | - | 17 (0.77) | 49 (0.61) |
| *JK*02W.03/02W.03.N211S* | - | - | - | - | - | - | - | - | - | - | 1 (0.12) | - | - | 1 (0.01) |
| *JK*02W.03/02W.03.P90S* | - | 2 (0.09) | - | - | - | - | - | - | 2 (1.23) | - | - | - | 4 (0.17) | 8 (0.10) |
| *JK*02W.03/02W.03.Y37X* | - | 1 (0.04) | - | - | - | - | - | - | - | - | - | - | - | 1 (0.01) |
| *JK*02W.03/02W.04.588A_G* | - | - | 1 (0.14) | - | - | 1 (0.42) | - | - | - | - | - | - | 1 (0.04) | 3 (0.04) |
| *JK*02W.03.M167V/02W.03.M167V* | - | - | - | - | 6 (3.85) | - | - | - | - | - | - | - | 1 (0.04) | 7 (0.09) |
| *JK*02W.03.M167V/02W.04.588A_G* | - | - | - | - | 1 (0.64) | - | - | - | - | - | - | - | - | 1 (0.01) |

^*^Details of allele names are given in Table 4. ^§^False genotyping possible, when restricted to JK1/JK2 determining SNV c.838G>A, ^$^False serological RBC phenotyping possible, depending on the reagents and protocols used. WEUR, Western Europe; TUR: Turkey; SYR, Syria; SSAF, Sub-Saharan Africa; SEEA, South East and East Asia; SAM, South America; NAF, Northern Africa; EEKCA, Eastern Europe, Caucasus and Central Asia; ARPE, Arabian Peninsula; OTH, Others (Country of origin not specified).
