# Supplementary material for: High-Throughput Next-Generation Sequencing of the Kidd Blood Group: Unexpected Antigen Expression Properties of Four Alleles and Detection of Novel Variants
Source: Transfus Med Hemother. 2022 Jul 26;50(1):51–65. doi: 10.1159/000525326 (PMC9911998; doi:10.1159/000525326)
Supplement: Supplementary file 6 — Supplementary data [file tmh-0050-0051-s06.docx]

Supplementary Table S6. Number of non-referenced alleles in various blood donor panels. Observation numbers were less than five.

| **Allele name^*^** | **WEUR (n=531)^§^** | **TUR (n=2,280)^§^** | **SYR (n=706)^§^** | **SSAF (n=70)^§^** | **SEEA (n=157)^§^** | **SAS (n=238)^§^** | **CSAM (n=78)^§^** | **NAF (n=227)^§^** | **IRAQ (n=167)^§^** | **IRAN (n=328)^§^** | **EEKCA (n=849)^§^** | **ARPE (n=195)^§^** | **OTH (n=2,207)** | **Total**  **(n=8,033)^§^** |
| --- | --- | --- | --- | --- | --- | --- | --- | --- | --- | --- | --- | --- | --- | --- |
| *JK*01.M352I* | - | 3 | - | - | - | - | - | - | - | 1 | - | - | - | 4 |
| *JK*01W.04.303G_A* | 1 |  | 1 | 1 |  |  |  |  |  |  |  |  | 1 | 4 |
| *JK*02W.03.V10M_V76I_303G_A* | - | 1 | 1 | 1 | - | - | - | - | - | - | - | - | 1 | 4 |
| *JK*01.L364F* | - | 1 | 1 | - | - | - | - | - | - | - | - | - | 1 | 3 |
| *JK*02W.03.840C_T* | 3 | 1 | - | - | - | - | - | - | - | - | - | - | - | 3 |
| *JK*02W.04.M167V_588A_G* | - | 2 | - | - | - | - | - | - | - | - | - | - | 1 | 3 |
| *JK*01W.01.D11N_588A_G* | 1 | - | - | - | - | - | - | - | - | 1 | - | - | 1 | 3 |
| *JK*01W.06.M167V* | - | - | - | - | 2 | - | 1 | - | - | - | - | - | - | 3 |
| *JK*01.105T_C* | - | 1 | 1 | - | - | - | - | - | - | - | - | - | - | 2 |
| *JK*01.159C_T* | 2 | - | - | - | - | - | - | - | - | - | - | - | - | 2 |
| *JK*01.E5+12T_C* | - | - | - | - | - | - | 1 | - | - | - | - | 1 | - | 2 |
| *JK*01.E4+10C_T* | - | 2 | - | - | - | - | - | - | - | - | - | - | - | 2 |
| *JK*01W.01.516C_T_588A_G* | - | 2 | - | - | - | - | - | - | - | - | - | - | - | 2 |
| *JK*01W.06.V54M* | - | - | - | - | - | - | - | 2 | - | - | - | - | - | 2 |
| *JK*02N.08.588A_G* | - |  | - | - | - | - | - | - | - | - | - | - | 1 | 1 |
| *JK*02W.03.957G_A* | - | - | - | - | - | - | - | - | - | - | - | 1 | 1 | 2 |
| *JK*02W.03.E371K* | - | - | - | - | - | - | - | - | - | - | 2 | - | - | 2 |
| *JK*02W.03.G325S* | - | - | 1 | - | - | - | - | - | - | 1 | - | - | - | 2 |
| *JK*01.163delG* | - | 1 | - | - | - | - | - | - | - | - | - | - | - | 1 |
| *JK*01.E5-19T_A* | - | - | 1 | - | - | - | - | - | - | - | - | - | - | 1 |
| *JK*01.E8+31G_A* | - | - | - | 1 | - | - | - | - | - | - | - | - | - | 1 |
| *JK*01.E9+18T_C* | - | - | 1 | - | - | - | - | - | - | - | - | - | - | 1 |
| *JK*01.E9-13T_C* | - | - | - | - | - | - | - | - | - | - | - | - | 1 | 1 |
| *JK*01.G27R* | - | - | - | - | - | 1 | - | - | - | - | - | - | - | 1 |
| *JK*01.G96V* | - | - | - | - | - | - | - | - | - | - | - | - | 1 | 1 |
| *JK*01.L188F_561C_T* | 1 | - | - | - | - | - | - | - | - | - | - | - | - | 1 |
| *JK*01.N373I* | - | - | - | - | - | - | - | - | - | - | - | - | 1 | 1 |
| *JK*01.R64Q* | - | - | - | - | - | - | - | - | - | - | 1 | - | - | 1 |
| *JK*01.T308I* | - | - | - | - | - | - | - | - | - | - | - | - | 1 | 1 |
| *JK*01.T346M* | - | - | - | - | - | - | - | - | - | - | - | - | 1 | 1 |
| *JK*01.V367A* | - | - | - | - | - | - | - | - | - | - | - | 1 | - | 1 |
| *JK*01.V54M* | - | - | - | - | - | 1 | - | - | - | - | - | - | - | 1 |
| *JK*01.V76A* | - | - | - | - | - | - | - | - | - | - | - | - | 1 | 1 |
| *JK*01.V87I* | - | - | 1 | - | - | - | - | - | - | - | - | - | - | 1 |
| *JK*01.V8F* | - | 1 | - | - | - | - | - | - | - | - | - | - | - | 1 |
| *JK*01N.09.V175I* | - | - | - | 1 | - | - | - | - | - | - | - | - | - | 1 |
| *JK*01W.01.219C_T_588A_G* | - | - | - | - | - | - | - | - | - | - | - | - | 1 | 1 |
| *JK*01W.01.279T_C_588A_G* | - | - | - | - | 1 | - | - | - | - | - | - | - | - | 1 |
| *JK*01W.01.588A_G_E9-7T_C* | - | - | - | - | 1 | - | - | - | - | - | - | - | - | 1 |
| *JK*01W.01.F329V_588A_G* | - | - | - | - | - | 1 | - | - | - | - | - | - | - | 1 |
| *JK*01W.01.P205S_588A_G* | - | - | - | - | 1 | - | - | - | - | - | - | - | - | 1 |
| *JK*01W.01.T127I_588A_G* | - | - | - | - | - | - | - | - | - | - | - | - | 1 | 1 |
| *JK*01W.02.402T_C_588A_G_E5-24C_T* | - | - | - | - | - | - | - | 1 | - | - | - | - | - | 1 |
| *JK*02N.05.588A_G* | - | - | - | - | - | - | - | - | - | - | - | - | 1 | 1 |
| *JK*02N.06.588A_G* | - | - | - | - | 1 | - | - | - | - | - | - | - | - | 1 |
| *JK*02N.09.210G_A_588A_G* | - | - | - | - | - | - | - | - | - | - | - | - | 1 | 1 |
| *JK*02N.17.588A_G_1095T_C* | - | - | 1 | - | - | - | - | - | - | - | - | - | - | 1 |
| *JK*02N.17.588A_G_957G_A* | - | - | - | - | - | - | - | - | - | - | - | - | 1 | 1 |
| *JK*02W.03.1047C_T* | - | - | - | - | - | - | - | - | - | - | - | - | 1 | 1 |
| *JK*02W.03.56insA_549G_A* | - | - | - | - | - | - | - | - | - | - | - | - | 1 | 1 |
| *JK*02W.03.591C_T* | - | - | - | - | 1 | - | - | - | - | - | - | - | - | 1 |
| *JK*02W.03.667C_T* | - | - | - | - | - | - | - | - | - | - | - | - | 1 | 1 |
| *JK*02W.03.678A_T* | - | 1 | - | - | - | - | - | - | - | - | - | - | - | 1 |
| *JK*02W.03.69G_A* | 1 | - | - | - | - | - | - | - | - | - | - | - | - | 1 |
| *JK*02W.03.948C_G* | - | - | - | - | - | - | - | - | - | - | - | 1 | - | 1 |
| *JK*02W.03.D113E* | - | - | - | - | - | - | - | - | - | - | - | - | 1 | 1 |
| *JK*02W.03.E8-38C_G* | - | - | 1 | - | - | - | - | - | - | - | - | - | - | 1 |
| *JK*02W.03.E9+9G_T* | 1 | - | - | - | - | - | - | - | - | - | - | - | - | 1 |
| *JK*02W.03.F376L* | - | 1 | - | - | - | - | - | - | - | - | - | - | - | 1 |
| *JK*02W.03.I375V* | - | - | - | - | - | - | - | - | - | - | - | 1 | - | 1 |
| *JK*02W.03.K355N_E9+9G_T* | - | - | - | - | - | - | - | 1 | - | - | - | - | - | 1 |
| *JK*02W.03.L322F* | - | - | - | - | - | - | - | - | - | - | 1 | - | - | 1 |
| *JK*02W.03.M7V* | - | 1 | - | - | - | - | - | - | - | - | - | - | - | 1 |
| *JK*02W.03.N211S* | - | - | - | - | - | - | - | - | - | - | 1 | - | - | 1 |
| *JK*02W.03.P363H* | - | - | - | - | - | - | - | - | 1 | - | - | - | - | 1 |
| *JK*02W.03.T95I_V175I* | - | - | - | - | - | - | - | - | - | 1 | - | - | - | 1 |
| *JK*02W.03.V76I* | 1 | - | - | - | - | - | - | - | - | - | - | - | - | 1 |
| *JK*02W.03.V385M* | - | - | - | - | - | - | - | - | - | 1 | - | - | - | 1 |
| *JK*02W.03.W144R* | - | 1 | - | - | - | - | - | - | - | - | - | - | - | 1 |
| *JK*02W.03.Y37X* | - | 1 | - | - | - | - | - | - | - | - | - | - | - | 1 |

^*^Details of alleles are given in Table 4. ^§^Total number of sample. WEUR, Western Europe; TUR: Turkey; SYR, Syria; SSAF, Sub-Saharan Africa; SEEA, South East and East Asia; SAM, South America; NAF, Northern Africa; EEKCA, Eastern Europe, Caucasus and Central Asia; ARPE, Arabian Peninsula; OTH, Others (Country of origin not specified).
