# Supplementary figures and images for: High-Throughput Next-Generation Sequencing of the Kidd Blood Group: Unexpected Antigen Expression Properties of Four Alleles and Detection of Novel Variants
Source: Transfus Med Hemother. 2022 Jul 26;50(1):51–65. doi: 10.1159/000525326 (PMC9911998; doi:10.1159/000525326)

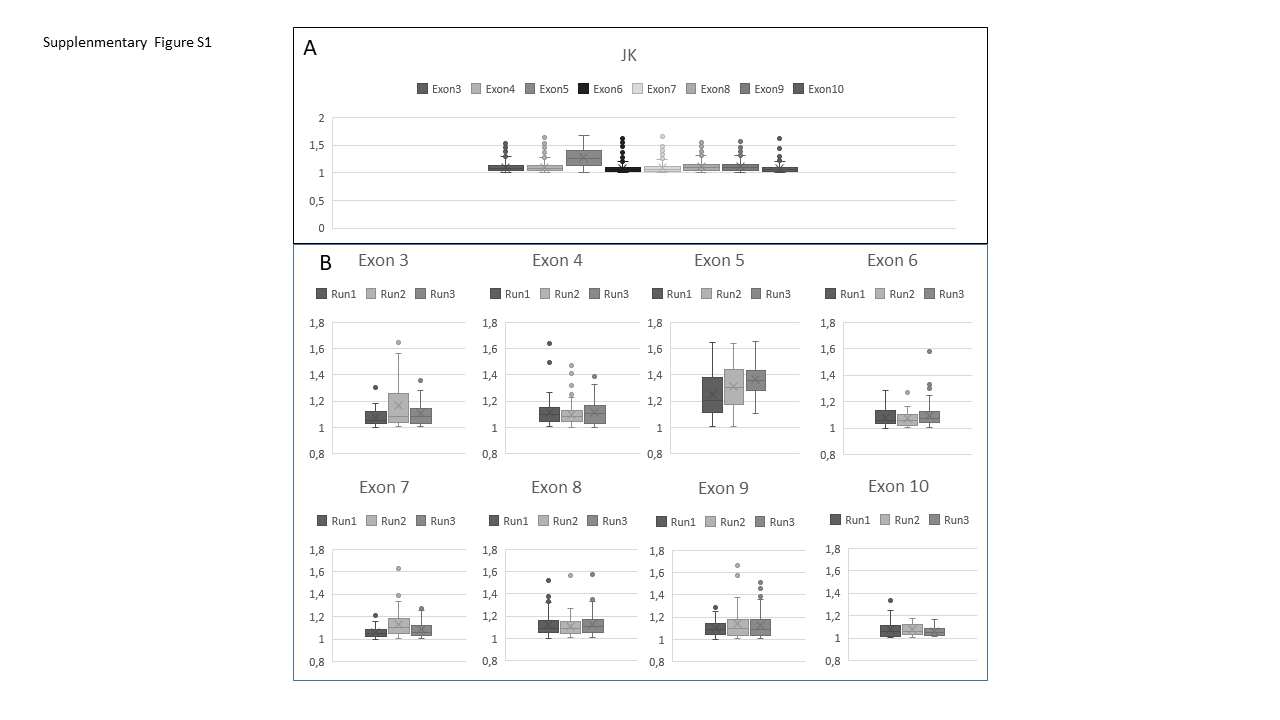

Supplement: Supplementary file 7 — Supplementary data [file tmh-0050-0051-s07.tif]
